# Delineating the trajectory of adult chronic diseases and healthcare use for 22q11.2 microdeletion in a general population context

**DOI:** 10.3389/fgene.2026.1737027

**Published:** 2026-02-13

**Authors:** Sarah L. Malecki, Tracy Heung, Samantha Morais, Refik Saskin, Drew Wilton, Therese A. Stukel, Eyal Cohen, Amol A. Verma, Anne S. Bassett

**Affiliations:** 1 General Internal Medicine Fellowship Program, University of Toronto, Toronto, ON, Canada; 2 Institute of Health, Policy Management and Evaluation, University of Toronto, Toronto, ON, Canada; 3 Child Health Evaluative Sciences and Department of Pediatrics, The Hospital for Sick Children, Toronto, ON, Canada; 4 Clinical Genetics Research Program, Centre for Addiction and Mental Health, Toronto, ON, Canada; 5 ICES, Toronto, ON, Canada; 6 Li Ka Shing Knowledge Institute & Department of Medicine, St Michael’s Hospital, University of Toronto, Toronto, ON, Canada; 7 Department of Psychiatry, University of Toronto, Toronto, ON, Canada; 8 Dalglish Family 22q Clinic, Division of Cardiology, & Toronto General Hospital Research Institute, University Health Network, Toronto, ON, Canada

**Keywords:** 22q deletion syndrome, accelerated aging, adult, cardiovascular outcomes, copy number variation, genetic disease, genetics-first, natural history

## Abstract

**Background:**

Children with complex genetic diseases increasingly survive to adulthood, but adult health is poorly understood. Using a genetics-first approach we investigated the incidence and accrual of cardiovascular and other outcomes in people with molecularly confirmed 22q11.2 microdeletion (22q-cases) compared with general population controls (population-comparators).

**Methods:**

Using a retrospective matched cohort study design, we linked 365 adult 22q-cases (median age 32 years; 51% female) to health administrative data for ∼15 million individuals with universal healthcare, identifying 3,650 well-matched population-comparators. We used Poisson regression to estimate incidence rate ratios (IRRs) and 95% CI for five cardiovascular/risk conditions and other outcomes, and recurrent event modelling to assess their relative rate (RR) of accrual over a median 28 years of retrospective and prospective health data.

**Results:**

Accrual of cardiovascular conditions occurred at a significantly greater relative rate (RR) in 22q-cases than population-comparators (RR 3.8, 95% CI 2.9–4.8; median ages 32, 31), even when restricting to 22q-cases with neither major congenital heart disease (CHD) nor schizophrenia (RR 3.6, 95% CI 2.4–5.4). Incidence was significantly greater in 22q-cases for hypertension and diabetes by age 18–24 (IRR 2.98, 95% CI 1.45–6.14; IRR 3.21, 95% CI 1.42–7.24, respectively), and by age 35–44 for heart failure. Other outcomes also showed increasing trajectories over young adult years in the 22q-case group, e.g., kidney disease, chronic obstructive pulmonary disease, healthcare resource use, and hospitalizations, including for individuals with neither CHD nor schizophrenia.

**Conclusion:**

A population-based approach provided new evidence for accumulating illnesses over young adulthood, supporting the need for novel models of anticipatory care for adults with 22q11.2 microdeletion. A similar genetics-first strategy, defining cohorts with shared genetic changes, may facilitate understanding of premature aging mechanisms relevant to the general population.

## Background

Copy number variations (CNVs), i.e., recurrent submicroscopic chromosomal deletions or duplications, represent a largely unrecognized genetic factor in adult disease development in the general population (Feuk et al., 2006; Crawford et al., 2019; Lupski et al., 2011; Martin et al., 2020; Birnbaum et al., 2022). CNVs may be considered as harbingers of the precision medicine revolution brought by molecular genetics to oncology, but with as yet limited impact on general adult medical practice. In part, this is because clinically-relevant (i.e., pathogenic) CNVs, are often not recognizable as clinically “syndromic” and are largely considered pediatric diseases, with clinical genetic testing implementation and phenotyping focused on infants and children, especially those with neurodevelopmental expression (Miller et al., 2010). Also, the pathogenicity, multisystem nature, and association with neurodevelopmental conditions of these CNVs contribute to marked under-ascertainment in large-scale adult biobanks, limiting understanding of their impact (Martin et al., 2020; Maleckiet al., 2024a; Bassett et al., 2023). A recent systematic review of recurrent deletion syndromes associated with pathogenic CNVs highlights the sparce incidence and prevalence data currently available for many important adult outcomes (Malecki et al., 2026).

The most common pathogenic CNV is the 22q11.2 microdeletion (22q) (McDonald-McGinn et al., 2015), with a live birth prevalence estimate of 1:2148, greater than that of cystic fibrosis (McDonald-McGinn et al., 2015; Blagojevic et al., 2021). One of many CNVs named for genotype rather than phenotype, 22q, with its multisystem manifestations, stands as a representative model for investigating how molecular genetic diagnosis relates to clinical complexity in young adults (Jonas et al., 2014; Malecki et al., 2020). For 22q, clinical genetic testing has been available since 1994, and advances in pediatric care have ensured that the vast majority of children with 22q survive to adulthood in first-world countries (McDonald-McGinn et al., 2015; Óskarsdóttir et al., 2023). While there are well-known associated features such as congenital cardiac or palatal anomalies and schizophrenia, comprehensive review of the literature has indicated only nascent data on adult health (Boot et al., 2023). There is evidence of premature adult mortality primarily related to cardiovascular causes, of elevated prevalence of chronic diseases like type 2 diabetes, and of high global health system costs for young adults (Van et al., 2019; Van et al., 2020; Malecki et al., 2024b). A formal review has revealed however that there are no data available on incidence trajectories of major diseases important to general adult health for 22q, or for other individual pathogenic deletion CNVs, compared to population-based controls (Malecki et al., 2025).

We aimed to examine trajectories of common chronic diseases in young adults, using 22q as a model CNV for a genetics or genotype-first strategy (Stessman et al., 2014), i.e., defining a cohort according to genetic etiology, in a population-based context. Linkage of a cohort comprising individuals with a molecularly confirmed 22q11.2 microdeletion to health administrative data for ∼15 million individuals living in Ontario with universal healthcare coverage (Malecki et al., 2024b) facilitated investigation of disease accrual, and of resource use trajectories. We focused on cardiovascular and related risk conditions as primary outcomes, given that these diseases represent leading non-infectious, non-traumatic causes of death in the general population (Statistics Canada, 2019-2022), and are measurable in administrative data (Moltó and Dougados, 2014). We hypothesized that across young adulthood there would be accelerated accrual of these conditions, and of healthcare resource use, among 22q-cases compared with matched population-based comparators, even in the absence of known 22q-associated conditions (congenital heart disease; schizophrenia) (McDonald-McGinn et al., 2015; Boot et al., 2023) that can contribute to mortality and to cardiovascular aging mechanisms (de Oliveira et al., 2016; Iacobazzi et al., 2022).

## Materials and methods

### Study design, setting and participants

This was a retrospective cohort study using Ontario health administrative data housed and accessed at ICES (formerly the Institute for Clinical Evaluative Sciences) (ICES, 2025), which captures all publicly insured healthcare services for the ∼15 million residents of Canada’s most populous province. See Supplementary Figure S1 and Supplementary Table S1 for study timelines showing data sources (Malecki et al., 2024b), and maximum data availability for the current study from 1 April 1988 to 31 August 2023. All study participants were required to be alive and Ontario residents as adults (age 18 years or older), eligible for publicly insured healthcare services at an arbitrary “index” date of 1 April 2002 representing inception of databases used to aggregate health system costs at ICES (Supplementary Figure S1) (Malecki et al., 2024b), and have basic demographic information available as at that date (date of birth, sex, and neighbourhood income quintile; Supplementary Table S1).

The study cohort comprised all individuals with a typical pathogenic 22q11.2 microdeletion molecularly-confirmed by clinical genetic testing, either fluorescence *in situ* hydridization using a targeted 22q11.2 region probe or genome-wide methods, e.g., microarray, from a broadly-ascertained clinical cohort of 365 adults. Nearly half of this cohort were first seen at age ≤21 years, i.e., at or about transition from pediatric care, and followed thereafter. Others were followed from time of referral/ascertainment from multiple sources, mainly adult cardiology, genetics, and psychiatry, with few (<10%) determined as a transmitting parent after the diagnosis of an affected offspring (as expected, given the known reduced reproductive fitness of 22q) (McDonald-McGinn et al., 2015; Malecki et al., 2020; Óskarsdóttir et al., 2023; Boot et al., 2023; Van et al., 2019; Van et al., 2020; Malecki et al., 2024b; Palmer et al., 2018; Palmer et al., 2022). These 365 individuals were linked to health administrative data held at ICES through Ontario Health Insurance Plan (OHIP) numbers (Malecki et al., 2024b). This cohort and linkage are described in detail elsewhere (Malecki et al., 2024b). All 22q-case participants provided informed consent and the study was approved by hospital research ethics boards.

The 22q-case cohort was *a priori* at the time of data linkage (January 2022) categorized into three clinically-defined subgroups (i.e., not based on health administrative codes) that were highly prevalent and could potentially affect ascertainment, cardiovascular risk, and health resource use (as described elsewhere) (Malecki et al., 2024b). The 22q-case subgroups designated were: 1) “Major congenital heart disease (CHD)” comprising all individuals born with moderate to severe CHD, e.g., tetralogy of Fallot, and with no psychotic illness; 2) “Schizophrenia” comprising individuals with major psychotic illness, mostly schizophrenia, and 3) “Neither” comprising individuals with neither major CHD nor psychotic illness (Malecki et al., 2024b). Certain other prevalent 22q features, such as intellectual disability (ID)/learning disability affects most individuals so was not considered as a subgroup (McDonald-McGinn et al., 2015; Malecki et al., 2020; Óskarsdóttir et al., 2023; Boot et al., 2023; Van et al., 2019; Van et al., 2020; Malecki et al., 2024b; Palmer et al., 2018; Palmer et al., 2022; Voll et al., 2017).

For each 22q-case, ten “unexposed” population-based comparators were selected, as described in detail elsewhere (Malecki et al., 2024b). Briefly, any individual meeting initial inclusion requirements, but neither in the 22q-case cohort nor having an administrative code that could indicate a 22q11.2 microdeletion or similar chromosomal condition, was eligible for selection as a comparator (Malecki et al., 2024b). These administrative code criteria were used because genetic data are not available in ICES which could be used to investigate the CNV carrier status. The comparator sample was selected based on greedy nearest neighbor matching using exact date of birth, sex, and neighbourhood income quintile at index as a proxy for socioeconomic status (Public Health Ontario, 2013).

Characteristics and outcomes were tabulated for 22q-cases and comparators over the entire observation (look-back/follow-up) period (maximum possible 35.4 years, Supplementary Figure S1): start of ICES data (1 April 1988), from birth, or start of OHIP coverage (e.g., by moving to Ontario), until death, loss of OHIP coverage, or latest follow-up date for data availability (31 August 2023) (see Supplementary Table S1).

### Variables

Age, sex, neighbourhood income quintile, and median years of follow-up were reported/compared for 22q-cases, 22q-case subgroups, and comparators, with standardized differences >0.10 indicative of significant imbalance between 22q-cases and comparators (Austin, 2009).

We defined primary outcomes for this study to be five cardiovascular/cardiovascular risk conditions included in the standard Charlson comorbidity index (Charlson et al., 1987) that had validated administrative data algorithms available (see supplement for validation study references): hypertension, diabetes, acute coronary syndromes including myocardial infarction (MI), heart failure, and stroke/transient ischemic attack (TIA). Incidence was defined as the first recorded instance of the condition, with the incidence rate per 1,000 person-years of each condition across five age groups: pediatric (0–17 years), and four adult age groups: 18–24, 25–34, 35–44, and 45+ years, tabulated from the beginning of the lookback period (i.e., start of ICES data in 1988, birth or start of OHIP coverage) until study end (Supplementary Figure S1).

Secondary outcomes included the incidence of two cardiovascular/risk conditions without validated algorithms (chronic kidney disease (CKD), peripheral vascular disease), the other seven chronic conditions from the Charlson index (chronic obstructive pulmonary disease (COPD), dementia, cancer, connective tissue diseases, peptic ulcer disease, liver disease, and HIV), and six conditions (intellectual disability, epilepsy, psychotic illness, scoliosis, hernia, hypothyroidism) known to be associated with 22q (McDonald-McGinn et al., 2015; Boot et al., 2023) (Supplementary Tables S2,S3). For conditions without a validated case definition, we used a standard algorithm applied to study adult multimorbidity (Supplementary Table S2) (Malecki et al., 2024b).

We also examined healthcare cost trajectories (extending our previous study that focused on overall adult healthcare costs) (Malecki et al., 2024b), using a standard Ontario case-costing methodology (Wodchis et al., 2013). Briefly, the case-costing algorithm was used from costing algorithm inception, i.e., an arbitrary index date (1 April 2002) to study end (latest, 31 August 2023; Supplementary Figure S1) to estimate person-level direct healthcare costs across all public healthcare sectors each year. Costs were inflated to 2023 dollars so they could be summed across years, then divided by person-years of follow-up. Median costs per person-year of follow-up were calculated and reported for the five age groups of interest. Sensitivity analyses included: 1) an alternative study end date (31 March 2020) to test the robustness of effects to health service disruptions during the pandemic, and 2) stratification by three “time since molecular diagnosis” groups (before diagnosis, in the first 5 years, >5 years since diagnosis). For this adult cohort, these time since diagnosis groups were selected to be consistent with previous studies that have reported increased healthcare costs in children 5 years after genetic diagnosis (Marshall et al., 2019).

In addition, we tabulated the cumulative number of adult admissions (at acute care and psychiatric hospitals) for each individual over the observation period (Supplementary Figure S1). We also report and compare mortality and median age at death for cases and comparators over the study period, as descriptive context for other outcomes.

### Statistical analysis

We examined the accrual of conditions within the composite group of five validated cardiovascular risk conditions/outcomes using recurrent event modelling. We used the Anderson-Gill Marginal Means model with age as the timescale (Andersen, 1993; Sutradhar et al., 2016) to model the relative rate (RR) and 95% confidence interval (CI) of accrual of the five composite conditions in adulthood for 22q11.2 microdeletion cases overall, and for each clinical subgroup, versus the overall matched population-comparators.

The incidence of individual chronic conditions over time was plotted and compared for 22q11.2 microdeletion cases overall, and for the “Neither” subgroup (i.e., without major CHD or schizophrenia), vs. overall matched population-based comparators, by calculating incidence rate ratios (IRRs). Analysis was performed at the level of the individual. Univariate Poisson regression models were used to generate effect estimates for the main exposure (22q or 22q subgroup) and 95% CI for each IRR. Overdispersion was addressed by scaling of standard errors, and the log of person-years at risk was used as an offset parameter. When the reported number of new cases violated reporting requirements at ICES (e.g., small (1–5) cell size or risk of back-calculation), a range of values was given and the midpoint number of cases and corresponding confidence intervals were plotted/used. For the composite cluster of validated cardiovascular conditions, conservative uncertainty intervals in the number of cases were used to estimate the incidence rate and IRR, based on the minimum and maximum number of cases.

Total healthcare costs were compared for 22q cases overall vs. matched comparators using generalized linear regression (Malecki et al., 2024b; Thavorn et al., 2017; Manning and Mullahy, 2001). A Tweedie distribution was used instead of a gamma distribution because of better fit with the age-stratified cost data. The natural logarithm was calculated from the Tweedie generalized linear model (log-link) and exponentiated coefficients were used to estimate the relative ratio (RRa) and 95% CI of costs for cases vs. comparators. Models were adjusted for length of follow-up as an offset term. Models were also run to compare the three 22q11.2 microdeletion subgroups (major CHD, schizophrenia, and neither) to the population-comparators overall. Sensitivity analyses using other (e.g., time since molecular diagnosis) subgroups were performed the same way.

The RR and 95% CI of adult hospitalizations for the 22q11.2 microdeletion case group overall, and for 22q11.2 microdeletion subgroups, versus overall comparators was estimated using recurrent event modelling. The Anderson-Gill Marginal Means model was used with age as the timescale (Andersen, 1993; Sutradhar et al., 2016).

We purposefully did not match/adjust beyond long-standing biologically immutable or early onset variables, e.g., age, sex and income quintile at costing index, and follow-up time (that is inherently variable), given that the exposure (i.e. 22q) is a genetic condition present since birth and many variables/conditions that occur later in life may be mediators on the causal pathway between exposure and outcome. To indicate meaningful differences, we used standardized differences >0.1 as a rule of thumb for descriptive variables (Austin, 2009), and effect sizes with 95% CI different from 1.0 to indicate statistical significance for the models tested, instead of p-values, as recommended for such analyses (Goodman, 2008). All analyses were perfomed in SAS enterprise guide v8.3 (SAS Institute).

## Results

Demographic characteristics and pediatric and adult years of follow-up for the 365 22q-cases and 3650 population-based comparators are summarized in Table 1. As a result of matching on sex and date of birth, the proportion of females (51% for both groups), and age at last follow-up (32 vs. 31 years, respectively), were well balanced. Study groups were also well balanced on median number of years of adult follow-up (14.6 for 22q vs. 13.6 for comparators), though 22q-cases had on average more pediatric years of follow-up, and thus more overall follow-up years, than population-comparators (Table 1). Supplementary Table S4 shows comparable data for each of the three 22q-case subgroups. By last follow-up, for the lowest income quintile, the proportion of population-comparators was lessened while little changed for 22q-cases, with more minor shifts from original distributions for other quintiles for both groups (Table 1).

**TABLE 1:** Demographic characteristics and years of healthcare data availability.

​	22q-cases		Population-comparators		Std Diff^a^
​	n	%	n	(%)	​
Female sex, n (%)	186	50.96%	1,860	50.96%	0.00
Income quintile at last follow-up, n (%)^b^					
1-low	98	26.85%	762	20.88%	**0.14**
2	66	18.08%	748	20.49%	0.06
3	67	18.36%	679	18.60%	0.01
4	67	18.36%	696	19.07%	0.02
5-high	61	16.71%	690	18.90%	0.06
Missing	6	1.64%	75	2.05%	0.03
Age group (y) at last follow-up, n (%)^d^					
00–17 (pediatric years)	0	0.00%	^c^1–5	-	c0.01–0.04
18–24	79	21.64%	777	21.29%	0.01
25–34	131	35.89%	1,385	37.95%	0.04
35–44	81	22.19%	^c^730–734	-	c0.05–0.10
45+	74	20.27%	757	20.74%	0.01
Years of healthcare (HC) data availability and age at last follow-up^d^					
Median total years of HC data (IQR)	28.90	(23.60–33.40)	25.40	(19.00–32.90)	**0.45**
Median pediatric years of HC data (IQR)	16.40	(5.60–18.00)	11.20	(0.00–18.00)	**0.31**
Median adult (≥18) years of HC data (IQR)	14.60	(8.00–24.20)	13.55	(7.80–24.00)	0.02
Median age (y) at last follow-up (IQR)	32.0	(25.0–42.0)	31.0	(25.0–41.0)	0.02

^a^ Std Diff = Standardized Difference; bold font indicates values >0.10, indicative of substantial imbalance between 22q-cases and population-based comparators.

^b^ Cases and comparators were matched on date of birth, sex, and income quintile as at the arbitrary “index” date of 1 April 2002, with proportions for both groups identical, as follows for quintiles 1 to 5, respectively: 25.48%, 20.55%, 17.53%, 17.53%, 18.90%, and with no missing data. Income quintiles at last follow-up indicate shifts over time/with aging, mainly in a decrease for comparators in quintile 1, as well as some decreases from quintiles 5 and 2 for the 22q-cases, and a new category of missing data, thus the Std Diff is no longer 0.00 for each, and shows a meaningful imbalance for the lowest quintile.

^c^ Due to ICES, policies prohibiting the release of small cell data, cell counts with values 1 to 5, or otherwise to avoid back-calculation, had data suppressed.

^d^ The maximum possible Ontario healthcare data availability in this study is for a 35.4 years time-span. All 22q participants were required to be adults and eligible for Ontario healthcare at time of data linkage in January 2022, and comparators with OHIP coverage as adults were matched to cases by exact date of birth. While more years of pediatric data, thus more total years of data, were available for the 22q-cases than comparators, the groups remain well-matched on median adult years of data, and on age and age-groups at last follow-up.

**FIGURE 1:**
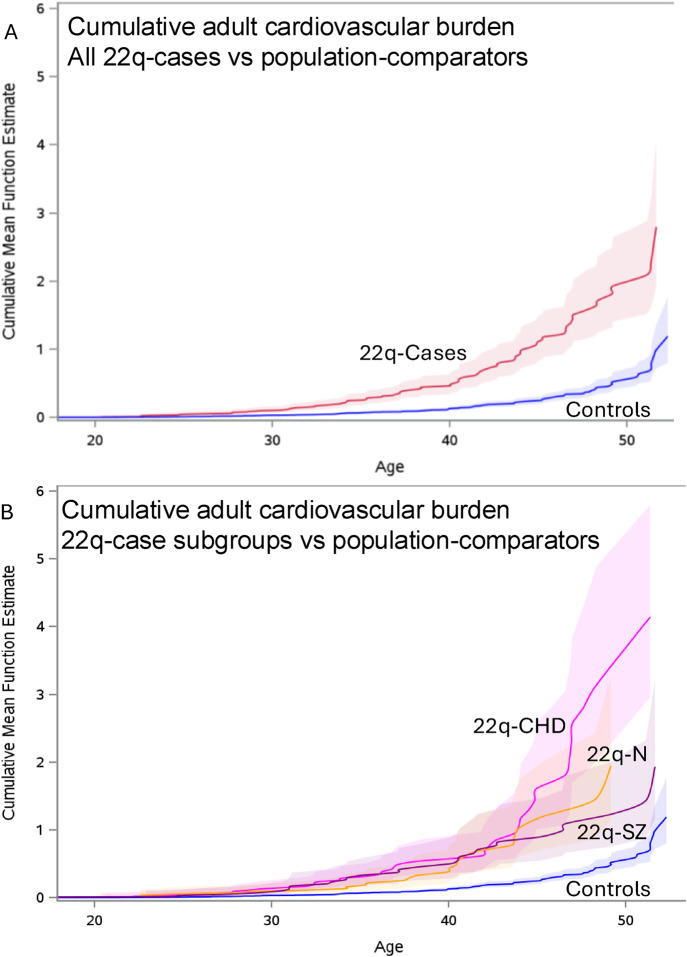
Cumulative cardiovascular outcome burden over adult years. Displayed are the plots with surrounding 95% CIs (paler colours) of mean cumulative numbers (Y-axis) over adult (≥18 years) ages (X-axis) of the five cardiovascular outcomes with validated case definitions available in health administrative data for all individuals with data available at age 18. These comprised two risk conditions (hypertension and diabetes) and three diseases (acute coronary syndromes, heart failure, and stroke/TIA). **(A)** shows the plots for all 22q-cases (“22q-Cases”; red) and population-comparators (“Controls”; blue). **(B)** shows the plots for each of three 22q-case subgroups: major CHD (“22q-CHD”; pink), schizophrenia (“22q-SZ”; purple), neither major CHD nor schizophrenia (“22q-N”; yellow), and the same population-comparator results (“Controls”; blue) as in **(A)**. Although the maximum possible number of conditions per individual is 5, upper 95% confidence intervals could include figures >5. The counting process was used to program the start and stop time; the SAS procedure PHREG was used to obtain the cumulative mean function to be plotted.

As hypothesized, accrual of the five cardiovascular risk conditions and diseases occurred in young adults with 22q at a greater relative rate than in population-based comparators [RR 3.8, 95% CI 2.9–4.8; Figure 1, Supplementary Table S5]. Similar findings were observed when the comparison was limited to the 22q-subgroup with neither major-CHD nor schizophrenia (RR 3.6, 95% CI 2.4–5.4).

**FIGURE 2:**
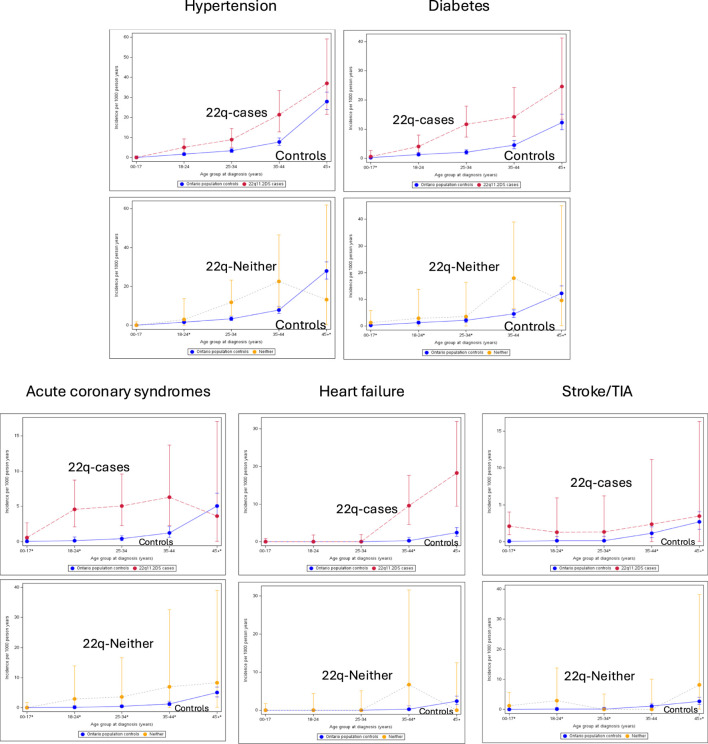
Incidence of individual cardiovascular risk conditions and diseases. For each of five labelled cardiovascular conditions the graphs display the incidence (first occurrence of the condition) per 1,000 person-years (Y-axis) across five age categories (X-axis). Results for all 22q-cases (“22q-Cases”; red) and general population-comparators (“Controls”; blue) appear above those for the 22q-case subgroup with neither major CHD nor schizophrenia (“22q-Neither”; yellow) and the same general population-comparators (“Controls”; blue). An asterisk (*) beside the age group on the X-axis indicates that the incidence rate ratio and confidence intervals for that age group were estimated, due to ICES policies prohibiting the resease of small cell data (<6). See text for further details.

Results for each of the five cardiovascular conditions are presented in Figure 2 and in Supplementary Tables S6–S10. By age 18–24, incidence of hypertension in 22q-cases was 2.98 times (95% CI 1.45–6.14), and that of diabetes 3.21 times (95% CI 1.42–7.24), higher than that of population-comparators. In the absence of major CHD and schizophrenia, incidence in 22q surpassed that of comparators by age 25–34 for hypertension (IRR 3.60, 95% CI 1.70–7.50), and by age 35–44 for diabetes (IRR 3.94, 95% CI 1.68–9.27).

Incidence was significantly greater for 22q-cases vs. population-comparators by age 18–24 (IRR 15.4–77.0, 95% CI 5.2–608.1) for acute coronary syndromes/MI, by age 35–44 years for heart failure (IRR 18.8–93.9, 95% CI 6.42–733.3), and in childhood for stroke/TIA (IRR 14.8–73.9, 95% CI 5.0–583.4). However, for these three conditions, numbers were small, and there were no significant differences for the 22q-subgroup without schizophrenia or major CHD.

Additional incidence plots for non-primary outcomes provide further exploratory results relevant to multi-system complexity in young adults with 22q11.2 microdeletions (Supplementary Figures S2,S3). For the other nine Charlson index conditions, several diseases of aging (CKD, COPD, and dementia) showed similar trajectories to those for hypertension, diabetes and heart failure (Supplementary Figure S2). In contrast, the incidence of all six 22q-related conditions studied here, including psychotic illness and hypothyroidism, diverged significantly in 22q-cases from population-comparators before age 18 as illustrated by non-overlapping confidence intervals, though each showed varying patterns of incidence results over the young adult age range (Supplementary Figure S3).

**FIGURE 3:**
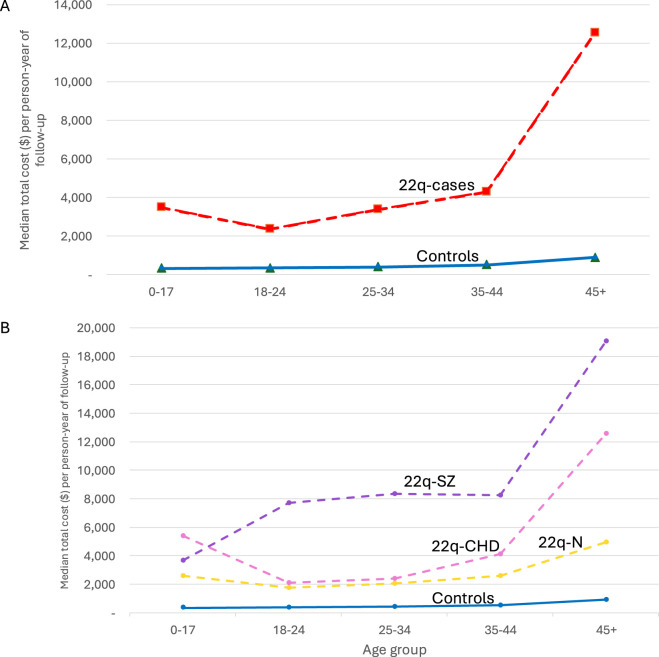
Healthcare cost trajectories. The plots display the median total cost per person-year of follow-up at each of five age categories (one pediatric, 0–17; four adult). **(A)** plots show all 22q-cases (“22q-Cases”; red) and general population-comparators (“Controls”; blue), and **(B)** plots show results for the three 22q-case subgroups: major CHD (pink), schizophrenia (purple), and neither major CHD nor schizophrenia (yellow), and for the same population-comparator results (blue) as in **(A)**.

Figure 3 depicts the median total healthcare cost per person-years of adult follow-up for each of five age groups. The relative ratio of costs for 22q vs. those for population-based comparators was greater for each age group, with the greatest difference, 10-fold greater for 22q, at age 35–44 years (RRa 10.0, 95% CI 8.3–12.2) (Supplementary Table S11). Cost trajectories varied by clinical 22q-subgroup, but remained higher than the general population for all subgroups, with costs reaching peak difference, 5-fold greater for the 22q subgroup with neither major-CHD nor schizophrenia, at age 35–44 (RR 5.1, 95% CI 3.7–6.9; Figure 3; Supplementary Table S12). Results were similar when restricting to a pre-pandemic time-frame, i.e., ending March 2020 (Supplementary Tables S13,S14). Stratifying by time since molecular genetic diagnosis did not eliminate significant associations of 22q with increased healthcare costs (Supplementary Table S15), and the overall trend of costs with age appeared similar between time since molecular diagnosis subgroups (Supplementary Figure S4).

**FIGURE 4:**
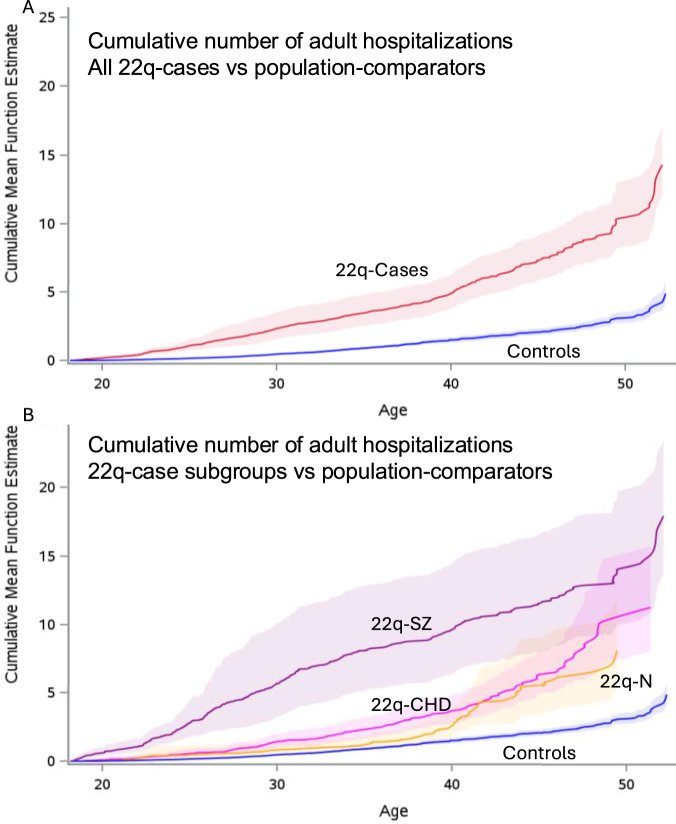
Cumulative adult hospitalizations. The graphs display the mean cumulative number of hospitalizations with surrounding 95% CIs (paler colours) (Y-axis) over adult (≥18 years) ages (X-axis) for all individuals with data available at age 18. **(A)** shows all 22q-cases (red) and population-comparators (blue), and **(B)** shows results for each of the three 22q-case subgroups: major CHD (“22q-CHD”; pink), schizophrenia (“22q-SZ”; purple), neither major CHD nor schizophrenia (“22q-N”; yellow), and the same population-comparator results (“Controls”; blue) as in **(A)**. Hospitalizations include both admissions to acute care (“DAD”) and psychiatric (“OMHRS”) hospitalizations. The counting process was used to program the start and stop time for hospitalizations, and the SAS procedure PHREG to obtain the cumulative mean function plotted.

The rate of adult hospitalizations for 22q-cases was significantly greater for 22q than for population-comparators (RR 3.9, 95% CI 3.6–4.2), including for 22q-cases with neither major-CHD nor schizophrenia (RR 2.1, 95% CI 1.8–2.4) (Figure 4; Supplementary Table S16).

Adult mortality was meaningfully different for 22q-cases compared to population-comparators (n = 38 deaths, 10.41%, vs. n = 65 deaths, 1.78%; standardized difference 0.37). Median age at death was on average 8 years younger for 22q-cases (median 43.0 years, IQR 27.0–54.0) than for population-comparators (51.0 years, IQR 41.0–61.0; standardized difference 0.52) who had died during follow-up, though with results varying for the three 22q-subgroups (Supplementary Table S4).

## Discussion

This study used a unique design to examine outcomes in adulthood: linking a genetically-defined complex disease model cohort to population-based administrative data for ∼15 million individuals with universal healthcare coverage (Malecki et al., 2024b). We used a lifespan approach with age-stratified analyses to focus on adult disease trajectories, substantially extending from our previous study that focused on cumulative healthcare costs and adding 3 years of follow-up data (Malecki et al., 2024b). The current study is the first to our knowledge to examine incidence and rate of accrual, i.e., the trajectories, of individual disease and other outcomes, across young adult age strata for individuals with a 22q11.2 microdeletion compared to matched population-based comparators. Consistent with our hypothesis, we found a significantly higher risk for, and rate of accumulation of, cardiovascular conditions of aging for 22q-cases in early adulthood, even in the absence of well-known predisposing factors. Chronic disease and resource use trajectories varied by 22q-subgroup, but acceleration of disease accrual and high healthcare needs in young adult years dominated the results for 22q-cases relative to population-based comparators.

Notably, individuals with 22q11.2 microdeletion but without either major CHD or schizophrenia were at increased risk of developing cardiovascular conditions. The adult trajectory of cardiovascular conditions in 22q-cases was, as expected, affected by the presence of major CHD (Figure 1), consistent with a recent review of cardiovascular aging in CHD (Iacobazzi et al., 2022), and for those in the 22q-case schizophrenia subgroup consistent with results for older individuals with schizophrenia in the general population (de Oliveira et al., 2016). These results support the possibility of additive or multiplicative effects of key 22q-phenotypes on outcomes and timing of cardiovascular disease. Collectively, the results suggest however that all individuals with 22q11.2 microdeletions should be monitored for cardiovascular risk in young adulthood beginning at transition to adult care, a new evidence-based finding and recommendation. Our age-stratified incidence rate data extend our understanding about timing of the increased risk of diabetes in 22q (Van et al., 2020), and provide new data on other risk conditions, e.g., hypertension. This evidence supports refinement in current guidance regarding cardiovascular and other disease risk screening for adults with 22q (Boot et al., 2023).

As expected, 22q11.2 microdeletion-associated conditions other than CHD and schizophrenia (i.e., intellectual disability, scoliosis, epilepsy, hypothyroidism) had elevated incidence before age 18 years, some of which may have facilitated molecular genetic diagnosis (at median age 11 in the cohort (Malecki et al., 2024b)). However, our results illustrate how long-term follow-up and population-based comparison can reveal diseases that are not otherwise known to be associated with the 22q11.2 microdeletion. Age-related diseases such as CKD, COPD, and dementia had similar risk profiles to the cardiovascular conditions, suggesting that accelerated aging may be a more global molecularly-defined phenomenon for this genetic condition (Kubben and Misteli, 2017). Clinical implications, supported also by the study results on healthcare use trajectories and early mortality, include targeted screening for diseases of aging in young adulthood to reduce the associated health burden and improve outcomes. Identifying genetic or other factors involved may lead to further opportunities to personalize care (Cleynen et al., 2021).

There is some evidence for accelerated cardiovascular aging in other genetic diseases (Donze et al., 2019; Hedgeman et al., 2017; Baksh et al., 2023), and in heterogeneous cohorts of CHD and intellectual disability likely to be enriched for genetic subtypes (Iacobazzi et al., 2022; Baksh et al., 2023). A systematic review has now provided evidence for between-CNV syndrome heterogeneity of cardiovascular risk diseases, supporting the likelihood that each genetic condition may have a unique risk profile (Malecki et al., 2025). A study of adults with Down syndrome provides a further concrete example, reporting reduced risk of hypertension and ischemic heart disease, yet increased risk of diabetes, obesity, and stroke (Baksh et al., 2023). In contrast, a United Kingdom biobank study involving healthy older adults (median age 65) reported that individuals with various CNVs (including just 10 with 22q11.2 microdeletion), considered collectively, had elevated prevalence of cardiovascular conditions compared to those without CNVs (Crawford et al., 2019). Evolving evidence of unique profiles of different genetic conditions suggests caution is needed when pooling such data, however (Malecki et al., 2025). Clearly, more adult data are needed to inform longitudinal care models for individual genetic conditions. Models focused broadly on adults with complex pediatric-onset diseases, beginning at transition to adult care and characterized by molecular genetic diagnoses, may be particularly valuable for targeted screening and management of cardiovascular conditions, and for identifying genetic subgroups with high risk profiles.

### Strengths and limitations

Strengths of this study include the use of a large, genetically-defined cohort, and linkage to population-based administrative data for individuals with universal healthcare coverage that facilitated capture of diseases and selection of well-matched comparators to account for basic demographic confounders. A long follow-up time to examine outcomes, and analyses within well-defined 22q subgroups, were further design advantages. There were also limitations. Selection bias for this study of adult illness trajectories includes survival to adulthood, i.e., could not include the estimated 4% (McDonald-McGinn et al., 2015; Óskarsdóttir et al., 2023) of children with severe manifestations dying before age 18 or others not in the 22q cohort studied. A fully representative cohort would require identification of all live births with 22q11.2 microdeletion and their follow-up through adult life. The high overall penetrance of this microdeletion, and the broad ascertainment of the cohort, including those transitioning from pediatric care or identified only through parental testing after diagnosis of an affected offspring, together with analyses of the 22q-subgroups, partially mitigate selection bias effects. No genetic testing was available for population-comparators, thus some individuals with a 22q11.2 microdeletion (expected <1/2000) could have been included, thereby affecting the results. However, the microdeletion’s rarity would suggest a negligible effect.

To define chronic diseases we used valid administrative algorithms where possible. All such algorithms vary in sensitivity and specificity and most have not been validated in young adults, thus could result in some misclassification. Subgroup analyses may suffer from type 2 error related to reduced statistical power, with low numbers in older age strata, e.g., of the 22q-subgroup with neither schizophrenia nor major CHD who were younger than the overall 22q-case cohort. All findings in this study, including estimates of disease risk, are conditional on being observed. Population-based comparators had fewer pediatric years of follow-up thus there may be less detection of chronic diseases in childhood, with this risk lessened however by these chronic conditions being recorded from age 18. Large effect sizes for many outcomes would have mitigated the chance of Type 1 error. Resource use may be under-estimated for the 22q-schizophrenia subgroup for years 2002–2006 when psychiatric hospital data were not available for the algorithm used, thus biasing towards the null. Onset of psychotic illness occurring in the <2 years after data linkage and before study end could have affected results for the clinically-determined major CHD and/or “Neither” subgroups that were designated just before data linkage. Variables not included in the linked datasets, e.g., reasons for initial clinical diagnosis or inherited (estimated 10%) vs. *de novo* microdeletion, could not be explored. The findings were robust to a sensitivity analysis stratifying by time since molecular diagnosis. However, age at molecular diagnosis would not necessarily reflect age at clinical diagnosis, and cohort effects related to lack of molecular testing prior to 1994.

## Conclusion

This study employed a genetics-first strategy to study long-term disease development for a genetically-defined condition with as yet limited data on adult phenotype, providing critical natural history data for adults with 22q11.2 microdeletion in a population context. Novel results suggest significantly higher risk of individual cardiovascular and other adult-onset conditions, and of the rate of accumulation of these conditions over time, even in the absence of well-known predisposing factors. The outcomes support the need for anticipatory screening for age-related diseases, and for novel models of care in adult medicine to optimally manage the accelerated clinical complexity − and increasing numbers (Malecki et al., 2024a) − of individuals with such genetically defined pediatric-onset diseases. Identification of 22q11.2 microdeletion in the general population could guide anticipatory care, early screening and preventive interventions to reduce adult disease burden. A population level genetics-first strategy focusing on improved implementation of genetic testing for at-risk adults, combined with seamless linkage of genetic testing data with administrative data, would facilitate comparisons of the risk of common adult-onset conditions among different genetically-defined cohorts. This may lead to discovery of mechanisms involved in premature aging, that could inform new treatment targets applicable to individuals in the general population (Malecki et al., 2024a).

## Data Availability

The dataset from this study is held securely in coded form at ICES. While data sharing agreements prohibit ICES from making the dataset publicly available, access may be granted to those who meet pre-specified criteria for confidential access, available at www.ices.on.ca/DAS. The full dataset creation plan and underlying analytic code are available from the authors upon request, understanding that the computer programs may rely upon coding templates or macros that are unique to ICES and are therefore either inaccessible or may require modification.
