# Supplementary material for: Delineating the trajectory of adult chronic diseases and healthcare use for 22q11.2 microdeletion in a general population context
Source: Front Genet. 2026 Feb 13;17:1737027. doi: 10.3389/fgene.2026.1737027 (PMC12945335; doi:10.3389/fgene.2026.1737027)
Supplement: Supplementary file 1 [file DataSheet1.pdf]

Supplement for “**Delineating the trajectory of adult chronic diseases and healthcare use for 22q11.2 microdeletion in a general population context**”

Sarah L. Malecki, Tracy Heung, Samantha Morais, Refik Saskin, Drew Wilton, Therese A. Stukel, Eyal Cohen, Amol A. Verma, Anne S. Bassett

Supplementary Tables S1-S16.
Supplementary Figures S1-S4.

Table S1. Data sources

Data source	Description of relevant data	Dates available for this study
Registered person data base (RPDB)	The RPDB provides basic demographic information (age, sex, location of residence, date of birth, and date of death for deceased individuals) for those issued an Ontario health insurance number. The RPDB also indicates the time periods for which an individual was eligible to receive publicly funded health insurance benefits and the best-known postal code for each registrant on July 1st of each year.	Apr 1991-Oct 2023
Postal code conversion file (PCCF)	The PCCF database will link to postal codes within a given cohort and determine other census geographic identifiers such as, dissemination/enumeration area, census division, longitude/latitude, urban/rural flag and neighbourhood income quintile.	Jan 1994-Dec 2022
Discharge Abstract Database (DAD)	The DAD is compiled by the Canadian Institute for Health Information and contains administrative, clinical (diagnoses and procedures/interventions), demographic, and administrative information for all admissions to acute care hospitals, rehab, chronic, and day surgery institutions in Ontario. At ICES, consecutive DAD records are linked together to form 'episodes of care' among the hospitals to which patients have been transferred after their initial admission.	Apr 1988-Aug 2023
Same day surgery (SDS)	The SDS is compiled by the Canadian Institute for Health Information and contains administrative, clinical (diagnoses and procedures), demographic, and administrative information for all patient visits made to day surgery institutions in Ontario. The main data elements include patient demographics, clinical data (diagnoses, procedures, physician), administrative data (institution/hospital number etc.), financial data, service-specific data elements for day surgery and emergency.	Apr 1991-Aug 2023
National Ambulatory Care Reporting System (NACRS)	The NACRS is compiled by the Canadian Institute for Health Information and contains administrative, clinical (diagnoses and procedures), demographic, and administrative information for all patient visits made to hospital- and community-based ambulatory care centres (emergency departments, day surgery units, hemodialysis units, and cancer care clinics). At ICES, NACRS records are linked with other data sources (DAD, OMHRS) to identify transitions to other care settings, such as inpatient acute care or psychiatric care.	ED Jul 2000-Oct 2023<br>Dialysis/Cancer Apr 2003-Oct 2023
Ontario health insurance plan (OHIP)	The OHIP claims database contains information on inpatient and outpatient services provided to Ontario residents eligible for the province's publicly funded health insurance system by fee-for-service health care practitioners (primarily physicians) and "shadow billings" for those paid through non-fee-for-service payment plans. The main data elements include patient and physician identifiers (encrypted), code for service provided, date of service, associated diagnosis, and fee paid.	Jul 1991-Oct 2023
Ontario Mental Health Reporting System (OMHRS)	The OMHRS is compiled by the Canadian Institute for Health Information and contains administrative, clinical (diagnoses and procedures), demographic, and administrative information for all admissions to adult designated inpatient mental health beds. This includes beds in general hospitals, provincial psychiatric facilities, and specialty psychiatric facilities. Clinical assessment data is ascertained using the Resident Assessment Instrument for Mental Health (RAI-MH), but different amounts of information are collected using this instrument depending on the length of stay in the mental health bed. Multiple assessments may occur during the length of a mental health admission.	Oct 2005-Aug 2023

Note, this includes major datasets used to define demographic variables and conditions, and is not a comprehensive overview of all datasets used to aggregate healthcare system costs. This can be found in Wodchis et al.[1]

Table S2. Cardiovascular disease and other Charlson condition sources

Condition	Algorithm/source	Sensitivity/Specificity (validation)	Codes/criteria (ICD-9, ICD-10)
**Cardiovascular conditions**			
Hypertension[a]	%HSPNmmb macro: One hospital admission (DAD/SDS) or two OHIP claims in two year period. (excluding claims 120 days before and 180 days after pregnancy admission)	For adults age 35+ (FP charts as gold standard): Sens (72%), Spec (95%), PPV (87%)<br>NPV (88%)[2]	ICD-9/OHIP dxcode: 401, 402, 403, 404, 405<br>ICD-10: I10, I11, I12, I13, I15
Diabetes[a]	%HSPNmmb macro:<br>**Adult definition:** two OHIP dxcode billings or one inpatient hospitalization or one ODB DM meds within a one-year period. (excluding OHIP and DAD claims 120 days before and 180 days after pregnancy admission)<br>**Pediatric definition**: used to determine DM prior to 19th birthday. four OHIP dxcode 250 claims or one OHIP feecode Q040, K029, K030, K045 and K046 claim within two years.	For adults age 19+:<br>Sens (86.1%), Spec (97.1%), PPV (79.8%), NPV (98.1%)[3]	ICD-9/OHIP dxcode: 250<br>ICD-10: E10, E11, E13, E14<br><br>OHIP feecode: Q040, K030, K029, K045, K046<br>Anti-diabetic drugs from ODB: subclass name = 'Oral Anti-Glycemics' or 'Insulin' or 'Antidiabetic agents: miscellaneous'
Chronic Kidney disease/Renal failure	%HSPNmmb: 1 DAD or 2+ OHIP in 2 years.[4]		ICD-9: 403, 404, 584, 585, 586, v451<br>ICD-10: N17, N18, N19, T82.4, Z49.2, Z99.2
Coronary syndrome (excluding MI)	%HSPNmmb: 1 DAD or 2+ OHIP in 2 years.[4]		ICD-9: 411-414<br>ICD-10: I20, I22-I25
Myocardial infarction (MI)[a]	%HSPNmmb macro: One DAD record with index admission/most responsible diagnosis of AMI	For adults age 21+, Sens 93%, Spec 89%[5]	ICD-9: 410<br>ICD-10: I21
Congestive heart failure[a]	%HSPNmmb macro: one hospital admission (either from the DAD or from OMHRS) with a CHF diagnosis or an OHIP claim/NACRS ED record with a CHF diagnosis followed within one year by either a second record with a CHF diagnosis from any source.	Case definition updated slightly compared to initial validation study: EMR (EMRALD for adults age 20+; other GPs for adults age 38+ as validation cohort): Sens (84.8%), Spec (97.0%), and PPV (55.3%)[6]	ICD-9/OHIP dxcode: 428<br>ICD-10: I500, I501, I509
Peripheral vascular disease	Standard algorithm: 1 DAD or 2+ OHIP in 2 years.[4]		ICD-9: 0930, 4373, 440, 441, 4431-4439, 4471, 5571, 5579, V434<br>ICD-10: I70, I71, I731, I738, I739, I771, I790, I792, K551, K558, K559, Z958, Z959<br>OHIP: 440,441
Stroke/TIA[a]	Valid case definition not available in HSPNmmb: One DAD record or two or more OHIP claims in 1 year.	Sens 68%, Spec 98.9%[7]	ICD-9: 362.3, 430, 431, 434.x, 436, and 435.x<br>ICD-10: I60.x, I61.x, I63.x (excluding I63.6 cerebral infarction due to central venous thrombosis),<br>I64, H34.1, and G45.x (excluding G45.4 transient global amnesia), H34.0<br>OHIP dxcode: 436, 432, 435

**Other Charlson conditions**			
COPD	%HSPNmmb macro: One COPD hospitalization in CIHI-SDS or CIHI-DAD COPD diagnosis, and/or three physician claim OHIP COPD records within two years.	Adults age 35+, primary care chart review by experts as gold standard: Sens (57.5%), Spec (95.4%), PPV (81.3%), and NPV (86.7%)[8]	ICD-9/OHIP dxcode: 491, 492, 496<br>ICD-10: J41, J42, J43, J44
Dementia	%HSPNmmb macro: One hospitalization (DAD/SDS) code OR (three physician claims codes at least 30 days apart in a two year period) OR a prescription filled for an AD-RD specific medication (ODB). [9]	Adults 65 and older, family practice EMR as gold standard: sensitivity 79.3%, specificity 99.1%, PPV 80.4%.[9]	ICD-9: 046.1, 290.0, 290.1, 290.2, 290.3, 290.4, 294, 331.0, 331.1, 331.5<br>ICD-10: F00, F01, F02, F03, G30<br>OHIP dxcode: 290, 331<br>Drug subclass: Cholinesterase inhibitors
CTD (RA/rheumatic like diseases, including osteoarthritis)	%HSPNmmb: 1 DAD or 2+ OHIP in 2 years.[4]		ICD9/OHIP dxcode:714, 715, 727, 729, 710, 720, 274, 716, 711, 718, 728, 739<br>ICD-10: M05-M06, M15-M19, M00-M03, M07, M10, M11-M14, M20-M25, M30-M36, M65-M79
Peptic ulcer disease	Standard algorithm: 1 DAD or 2+ OHIP in 2 years.[4]		ICD-9: 531-534<br>ICD-10: K25-K28<br>OHIP dxcode: 531, 532, 534
Liver disease (mild-severe)	Standard algorithm: 1 DAD or 2+ OHIP in 2 years.[4]		ICD-9: 0702, 0703, 0704, 0705, 0706, 0709, 570, 571, 5733, 5734, 5738, 5739, V427<br>4560, 4561, 4562, 5722, 5723, 5724, 5725, 5726, 5727, 5728<br>ICD-10: B18, K700-K703, K709, K713-K715, K717, K73, K74, K760, K762-K764, K768, K769, Z944<br>I850 I859 I864 I982 K704 K711 K721 K729 K765 K766 K767<br>OHIP: 571
HIV/AIDS	Standard algorithm: 1 DAD or 2+ OHIP in 2 years.[4]		ICD-9: 042-044<br>ICD-10: B20-B22, B24<br>OHIP: 042, 043, 044
Cancer	%HSPNmmb: 1 DAD or 2+ OHIP in 2 years.[4]		ICD-9:140-239<br>ICD-10: C00-C26, C30-C44, C45-C97

Some overlap with definitions for conditions reported in Malecki et al 2024.[10] Codes come from a standard multimorbidity measure at ICES (%HSPNmmb macro) which applies valid case definitions or a default algorithm[4] to define a condition. For CV/Charlson conditions not included in this standard multimorbidity measure (PVD, PUD, liver disease, HIV/AIDS) the standard Charlson index definition was used at ICES (includes ICD-9 and 10 codes and applied to inpatient data, and OHIP codes were added to increase sensitivity and apply a standard disease defintion.[4] a indicates CV conditions with a valid administrative case algorithm.

Table S3. 22q-associated condition definitions

Condition	Algorithm/source (* if validated)	Codes/criteria<br>ICD-9, ICD-10, other
Intellectual disability (ID), including Intellectual disability resulting from chromosomal anomalies, and other intellectual disabilities (e.g., fetal alcohol syndrome, tuberous sclerosis)	Two physician visits or at least one ED visit or hospitalization from database inception to last follow-up.[11]	ICD-10: F70-F73, F78, F79, Q90, Q91, Q92.0-Q92.5, Q92.7-Q92.9, Q93, Q97.1, Q99.2, Q99.8 Q85.1, Q86.1, Q87.1, Q87.23, Q87.31, Q87.8<br>ICD-9: 317-319, 758.0-758.3, 758.5, 758.8 (not 758.81, only if 5 digits exist), 758.9, 759.5<br>OHIP: 319<br>OMHRS: Q2d (i.e., Axis II) in 317, 318, 318.0, 318.1, 318.2, 319 (and retired fields).<br>OMHRS: I11h–I11m = any diagnosis of Qxxx as listed in ICD-10 column, Q3 = 1.
Scoliosis	Standard disease definition.	ICD-10: M41, Q67.5, M96.5, Q76.3<br>ICD-9: 737.3, 737.4, 754.2<br>OHIP: 737
Epilepsy	Define if the diagnostic code was recorded in 2 physician visits or at least 1 ED visit or hospitalization from database inception to last follow-up.[11]	ICD-10: G40<br>ICD-9: 345.0-345.1, 345.4-345.9<br>OHIP: 345
Hernia	Standard disease definition.	ICD-10: K40-K46<br>ICD-9: 550-553, 756.6<br>OHIP: 550, 552, 553
Psychotic illness	*Defined using previously defined algorithm in Kurdyak et al 2015[12] as a base (selected most sensitive algorithm with highest PPV): Use CIHI-DAD ICD-10 codes before 2005 and OMHRS after<br>2005. Define psychotic illness as 1 hospitalization or<br>2 MD visits in a 24- month period. We also added DSM-V diagnoses that were not available for the previous algorithm.	ICD-10: F20, F25, F29<br>ICD-9: 295, 298<br>OHIP: 295, 298<br>OMHRS after 2005: DSM- IV diagnoses of Schizophrenia, schizoaffective disorder or psychotic disorder NOS: 29510, 29520, 29530, 29570 and 29890.<br>https://www.ices.on.ca/publications/research-reports/mental-health-and-addictions-system-performance-in-ontario-2021-scorecard/ technical appendix: MHASEF old and new definition and general MHA diagnostic groups using DSM-IV, DSM-5 (OMHRS), ICD-10-CM (including and excluding provisional codes).
Hypothyroidism	1 hospitalization or 2 claims in 2 years or less.[13]	ICD-10: E00-E03, E89.0<br>ICD-9: 240.9, 243, 244, 246.1, 246.8<br>OHIP: 243, 244

Conditions were defined and reported as described in Malecki et al 2024.[10] Definitions repeated above. Excluding CHD and palate anomalies, as congenital.
Standard disease definition: One inpatient hospital diagnostic code or two or more outpatient physician billing codes in two years.[4]

Table S4. Baseline characteristics and mortality for 22q-cases, stratified by the three 22q-case subgroups

	**Schizophrenia subgroup**	**Major CHD subgroup**	**Neither schizophrenia nor major CHD subgroup**
	N=97	N=108	N=160
Median age in years at last follow-up (IQR)	40.0 (29.0-51.0)	31.0 (25.0-38.0)	28.5 (23.0-39.5)
00-17	0 (0.00%)	0 (0.00%)	0 (0.00%)
18-24	6 (6.19%)	22 (20.37%)	51 (31.88%)
25-34	33 (34.02%)	46 (42.59%)	52 (32.5%)
35-44	21 (21.65%)	30 (27.78%)	30 (18.75%)
45+	37 (38.14%)	10 (9.26%)	27 (16.88%)
Female sex, n (%)	44 (45.36%)	55 (50.93%)	87 (54.38%)
Income quintile at last follow-up, n (%)			
1 - low	30 (30.93%)	26 (24.07%)	42 (26.25%)
2	19 (19.59%)	21 (19.44%)	26 (16.25%)
3	16 (16.49%)	22 (20.37%)	29 (18.13%)
4	11 (11.34%)	23 (21.3%)	33 (20.63%)
5 - high	*16-20	*11-15	*25-29
Missing	*1-5	*1-5	*1-5
Median years of follow-up (IQR)	30.10 (25.80-33.40)	29.00 (25.00-33.40)	27.45 (22.60-33.40)
Median pediatric years of follow-up (IQR)	8.70 (0.00-18.00)	17.85 (11.10-18.00)	17.85 (7.85-18.00)
Median adult years of follow-up (IQR)	22.40 (11.90-33.20)	13.55 (7.55-20.35)	11.00 (5.95-22.20)
**Mortality**			
Number of deaths during the study period	19 (19.59%)	12 (11.11%)	7 (4.38%)
Median age in years at death (IQR)	45.0 (38.0-56.0)	27.0 (22.0-40.5)	54.0 (43.0-67.0)

CHD = congenital heart disease; IQR = inter-quartile range. * Small cell data suppressed as ICES policies prohibit the release of small cell data (<6) or other corresponding data allowing back calculation.

Table S5. Accrual of Cardiovascular Conditions

	Andersen-Gill/Marginal Means and Rates Model	
	**Crude RR (95% CI)**	**Crude p-value**
**Multimorbidities from 18 years on among those with follow-up**		
Population-based comparators	1 (Ref.)	
22q Cases	3.76 (2.93-4.82)	<0.0001
Schizophrenia	2.83 (1.90-4.22)	<0.0001
Major CHD	5.34 (3.78-7.55)	<0.0001
Neither	3.58 (2.40-5.36)	<0.0001

Conditions included as cardiovascular multimorbidities are myocardial infarction (MI), congestive heart failure (CHF), hypertension (HTN), diabetes (DM), and Stroke/ transient ischemic attack (TIA). See text and e-Table 2 for definitions.
Relative rate (RR) of accrual of multimorbidities was estimated using the recurrent event framework Andersen-Gill)/Marginal Means and Rates Model. The time scale used was age. See text for more details.
CI = confidence interval. CHD = congenital heart disease. Neither = neither major CHD nor schizophrenia.
Confidence intervals were calculated based on a robust variance estimator accounting for clustering at the level of the individual.
P-values for the relative rate estimates were calculated using the Wald chi-square statistic.

Table S6. Incidence of hypertension

Group	Age group	N at start of age interval	Median (IQR) age at diagnosis	New cases	PYAR	Incidence per 1000 person years	IR lower	IR upper	Incidence rate ratio (95%CI)	p-value	Incidence rate ratio (95%CI) if 1 or lower value of range provided	Incidence rate ratio (95%CI) if 5 or higher value of range provided
					Hypertension							
Ontario	00-17	2482	. (.-.)	0	35019.37	0.00	.	0.11	1 (Ref.)		1 (Ref.)	1 (Ref.)
Ontario	18-24	2902	22.0 (20.5-23.5)	28	16693.62	1.68	1.11	2.42	1 (Ref.)		1 (Ref.)	1 (Ref.)
Ontario	25-34	2814	30.0 (27.0-31.0)	53	15924.95	3.33	2.49	4.35	1 (Ref.)		1 (Ref.)	1 (Ref.)
Ontario	35-44	1516	39.0 (37.0-42.0)	71	9156.86	7.75	6.06	9.78	1 (Ref.)		1 (Ref.)	1 (Ref.)
Ontario	45+	757	52.0 (48.0-58.0)	166	5934.91	27.97	23.88	32.56	1 (Ref.)		1 (Ref.)	1 (Ref.)
Ontario	Total	3650	45.0 (34.0-53.0)	318	82729.71	3.84			1 (Ref.)		1 (Ref.)	1 (Ref.)
22q11.2DS	00-17	302	. (.-.)	0	4378.20	0.00	.	0.84	...	...		
22q11.2DS	18-24	333	22.5 (21.0-24.0)	10	1998.39	5.00	2.40	9.20	2.98 (1.45-6.14)	0.003		
22q11.2DS	25-34	292	31.0 (27.0-32.5)	16	1789.13	8.94	5.11	14.52	2.69 (1.54-4.70)	0.0005		
22q11.2DS	35-44	163	40.0 (38.0-41.0)	19	888.31	21.39	12.88	33.40	2.76 (1.66-4.58)	<.0001		
22q11.2DS	45+	74	51.0 (46.0-55.0)	17	460.30	36.93	21.51	59.13	1.32 (0.80-2.18)	0.275		
22q11.2DS	Total	365	38.0 (28.0-45.0)	62	9514.33	6.52			1.70 (1.29-2.23)	0.0001		
22q11.2DS - Neither	00-17	136	. (.-.)	0	2037.56	0.00	.	1.81	...	...		
22q11.2DS - Neither	18-24	147	*NA	*1-5	847.83	*1.18-5.90	.	.	*0.70-3.52 (0.10-9.11)	<0.06	0.70 (0.10-5.17)	3.52 (1.36-9.11)
22q11.2DS - Neither	25-34	113	31.0 (27.5-32.5)	8	677.55	11.81	5.10	23.27	3.55 (1.69-7.46)	0.0008		
22q11.2DS - Neither	35-44	62	40.0 (40.0-43.0)	7	309.51	22.62	9.09	46.60	2.92 (1.34-6.34)	0.0069		
22q11.2DS - Neither	45+	27	*NA	*1-5	188.34	*5.31-26.55	.	.	*0.19-0.95 (0.03-2.31)	<1	0.19 (0.03-1.36)	0.95 (0.39-2.31)
22q11.2DS - Neither	Total	160	37.0 (28.0-43.0)	24	4060.79	5.91			1.54 (1.02-2.33)	0.0421		

Ontario = Ontario population comparators. 22q11.2DS = 22q11.2 deletion syndrome, i.e. cases. 22q11.2DS – Neither = the subgroup of 22q with neither major congenital heart disease or schizophrenia. Age group is in years N=number. IQR = inter-quartile range. PYAR = person-years at risk, calculated as any risk time observed within the age group, even if beginning in the middle or towards the end of a period. IR = incidence rate. * Small cell data suppressed as ICES policies prohibit the release of small cell data (<6) or other corresponding data allowing back calculation. A confidence interval not including 1.0 was considered statistically significant. P-values for the incidence rate ratios were calculated using the Wald chi-square statistic.

Table S7. Incidence of acute coronary syndrome or myocardial infarction

Group	Age group	N at start of age interval	Median (IQR) age at diagnosis	New cases	PYAR	Incidence per 1000 person years	IR lower	IR upper	Incidence rate ratio (95%CI)	Incidence rate ratio (95%CI) if 1 or lower value of range provided	Incidence rate ratio (95%CI) if 5 or higher value of range provided	Incidence rate ratio (95%CI) if 22q have 1 or lower value of range provided, and controls have 5 or higher value of range provided	Incidence rate ratio (95%CI) if controls have 1 or lower value of range provided, and 22q have 5 or higher value of range provided
Coronary syndrome or acute myocardial infarction													
Ontario	00-17	2482	*NA	*1-5	35018.79	*0.03-0.14	.	.	1 (Ref.)	1 (Ref.)	1 (Ref.)	1 (Ref.)	1 (Ref.)
Ontario	18-24	2902	*NA	*1-5	16743.23	*0.06-0.30	.	.	1 (Ref.)	1 (Ref.)	1 (Ref.)	1 (Ref.)	1 (Ref.)
Ontario	25-34	2814	30.0 (27.0-33.0)	7	16233.30	0.43	0.17	0.89	1 (Ref.)	1 (Ref.)	1 (Ref.)	1 (Ref.)	1 (Ref.)
Ontario	35-44	1516	41.0 (39.5-43.0)	12	9685.34	1.24	0.64	2.16	1 (Ref.)	1 (Ref.)	1 (Ref.)	1 (Ref.)	1 (Ref.)
Ontario	45+	757	52.0 (48.0-62.0)	39	7727.32	5.05	3.59	6.90	1 (Ref.)	1 (Ref.)	1 (Ref.)	1 (Ref.)	1 (Ref.)
Ontario	Total	3650	47.0 (40.0-56.0)	62	85407.97	0.73			1 (Ref.)	1 (Ref.)	1 (Ref.)	1 (Ref.)	1 (Ref.)
22q11.2DS	00-17	302	*NA	*1-5	4327.24	*0.23-1.16	.	.	*1.62-40.46 (0.19-346.34)	8.09 (0.51-129.38)	8.09 (2.34-27.95)	1.62 (0.19-13.85)	40.46 (4.73-346.34)
22q11.2DS	18-24	333	20.0 (19.0-21.0)	9	1956.05	4.60	2.10	8.73	*15.41-77.04 (5.16-608.06)	77.04 (9.76-608.06)	15.41 (5.16-45.97)		
22q11.2DS	25-34	292	28.0 (27.0-30.0)	9	1781.96	5.05	2.31	9.59	11.71 (4.36-31.45)				
22q11.2DS	35-44	163	39.5 (37.0-40.0)	6	952.87	6.30	2.31	13.71	5.08 (1.91-13.54)				
22q11.2DS	45+	74	*NA	*1-5	684.84	*1.46-7.30	.	.	*0.29-1.45 (0.04-3.67)	0.29 (0.04-2.11)	1.45 (0.57-3.67)		
22q11.2DS	Total	365	27.0 (20.0-39.0)	33	9702.96	3.40			4.69 (3.07-7.15)				
22q11.2DS - Neither	00-17	136	. (.-.)	0	2037.56	0.00	.	1.81	...				
22q11.2DS - Neither	18-24	147	*NA	*1-5	840.70	*1.19-5.95	.	.	*3.98-99.58 (0.47-852.34)	19.92 (1.25-318.40)	19.92 (5.77-68.79)	3.98 (0.47-34.09)	99.58 (11.63-852.34)
22q11.2DS - Neither	25-34	113	*NA	*1-5	700.98	*1.43-7.13	.	.	*3.31-16.54 (0.41-52.12)	3.31 (0.41-26.89)	16.54 (5.25-52.12)		
22q11.2DS - Neither	35-44	62	*NA	*1-5	358.22	*2.79-13.96	.	.	*2.25-11.27 (0.29-31.98)	2.25 (0.29-17.33)	11.27 (3.97-31.98)		
22q11.2DS - Neither	45+	27	*NA	*1-5	298.95	*3.35-16.73	.	.	*0.66-3.31 (0.09-8.41)	0.66 (0.09-4.82)	3.31 (1.31-8.41)		
22q11.2DS - Neither	Total	160	34.0 (19.0-50.0)	6	4236.41	1.42			1.95 (0.84-4.51)				

Ontario = Ontario population comparators. 22q11.2DS = 22q11.2 deletion syndrome, i.e. cases. 22q11.2DS – Neither = the subgroup of 22q with neither major congenital heart disease or schizophrenia. Age group is in years N=number. IQR = inter-quartile range. PYAR = person-years at risk, calculated as any risk time observed within the age group, even if beginning in the middle or towards the end of a period. IR = incidence rate. * Small cell data suppressed as ICES policies prohibit the release of small cell data (<6) or other corresponding data allowing back calculation. A confidence interval not including 1.0 was considered statistically significant.

Table S8. Incidence of congestive heart failure

Group	Age group	N at start of age interval	Median (IQR) age at diagnosis	New cases	PYAR	Incidence per 1000 person years	IR lower	IR upper	Incidence rate ratio (95%CI)	p-value	Incidence rate ratio (95%CI) if 1 or lower value of range provided	Incidence rate ratio (95%CI) if 5 or higher value of range provided	Incidence rate ratio (95%CI) if 22q have 1 or lower value of range provided, and controls have 5 or higher value of range provided	Incidence rate ratio (95%CI) if controls have 1 or lower value of range provided, and 22q have 5 or higher value of range provided
				Congestive heart failure										
Ontario	00-17	2482	. (-.)	0	35019.37	0.00	.	0.11	1 (Ref.)		1 (Ref.)	1 (Ref.)	1 (Ref.)	1 (Ref.)
Ontario	18-24	2902	. (-.)	0	16756.20	0.00	.	0.22	1 (Ref.)		1 (Ref.)	1 (Ref.)	1 (Ref.)	1 (Ref.)
Ontario	25-34	2814	. (-.)	0	16278.98	0.00	.	0.23	1 (Ref.)		1 (Ref.)	1 (Ref.)	1 (Ref.)	1 (Ref.)
Ontario	35-44	1516	*NA	*1-5	9778.31	*0.10-0.51	.	.	1 (Ref.)		1 (Ref.)	1 (Ref.)	1 (Ref.)	1 (Ref.)
Ontario	45+	757	61.0 (59.0-69.5)	20	8243.69	2.43	1.48	3.75	1 (Ref.)		1 (Ref.)	1 (Ref.)	1 (Ref.)	1 (Ref.)
Ontario	Total	3650	61.0 (59.0-69.0)	*21-25	86076.55	*0.24-0.29			1 (Ref.)		1 (Ref.)	1 (Ref.)	1 (Ref.)	1 (Ref.)
22q11.2DS	00-17	302	. (-.)	0	4378.20	0.00	.	0.84	...	...				
22q11.2DS	18-24	333	. (-.)	0	2017.46	0.00	.	1.83	...	...				
22q11.2DS	25-34	292	. (-.)	0	1920.27	0.00	.	1.92	...	...				
22q11.2DS	35-44	163	40.5 (40.0-44.0)	10	1041.74	9.60	4.60	17.65	*18.77-93.87 (6.42-733.25)	<.0001	93.87 (12.02-733.25)	18.77 (6.42-54.92)		
22q11.2DS	45+	74	49.5 (46.5-55.5)	12	655.21	18.31	9.46	31.99	7.55 (3.69-15.44)	<.0001				
22q11.2DS	Total	365	46.0 (41.0-50.0)	22	10012.87	2.20			*7.56-9.01 (4.27-16.38)	<.0001	9.01 (4.95-16.38)	7.56 (4.27-13.42)		
22q11.2DS - Neither	00-17	136	. (-.)	0	2037.56	0.00	.	1.81	...	...				
22q11.2DS - Neither	18-24	147	. (-.)	0	852.53	0.00	.	4.33	...	...				
22q11.2DS - Neither	25-34	113	. (-.)	0	721.22	0.00	.	5.11	...	...				
22q11.2DS - Neither	35-44	62	*NA	*1-5	368.97	*2.71-13.55	.	.	*5.30-132.51 (0.62-1134.19)	<0.05	26.50 (1.66-423.7)	26.50 (7.67-91.54)	5.30 (0.62-45.37)	132.51 (15.48-1134.19)
22q11.2DS - Neither	45+	27	. (-.)	0	294.90	0.00	.	12.51	...	...				
22q11.2DS - Neither	Total	160	*NA	*1-5	4275.18	*0.23-1.17			*0.81-4.79 (0.11-1.81)	<1	0.96 (0.13-7.13)	4.03 (1.54-10.52)	0.81 (0.11-5.94)	4.79 (1.81-12.71)

Ontario = Ontario population comparators. 22q11.2DS = 22q11.2 deletion syndrome, i.e. cases. 22q11.2DS – Neither = the subgroup of 22q with neither major congenital heart disease or schizophrenia. Age group is in years N=number. IQR = inter-quartile range. PYAR = person-years at risk, calculated as any risk time observed within the age group, even if beginning in the middle or towards the end of a period. IR = incidence rate.
* Small cell data suppressed as ICES policies prohibit the release of small cell data (<6) or other corresponding data allowing back calculation.
A confidence interval not including 1.0 was considered statistically significant. P-values for the incidence rate ratios were calculated using the Wald chi-square statistic.

Table S9. Incidence of diabetes

Group	Age group	N at start of age interval	Median (IQR) age at diagnosis	New cases	PYAR	Incidence per 1000 person years	IR lower	IR upper	Incidence rate ratio (95%CI)	p-value	Incidence rate ratio (95%CI) if 1 or lower value of range provided	Incidence rate ratio (95%CI) if 5 or higher value of range provided
						Diabetes						
Ontario	00-17	2482	12.5 (7.0-14.0)	10	34951.85	0.29	0.14	0.53	1 (Ref.)		1 (Ref.)	1 (Ref.)
Ontario	18-24	2902	22.0 (20.0-23.0)	21	16655.97	1.26	0.78	1.93	1 (Ref.)		1 (Ref.)	1 (Ref.)
Ontario	25-34	2814	30.0 (29.0-32.0)	34	16077.29	2.11	1.46	2.96	1 (Ref.)		1 (Ref.)	1 (Ref.)
Ontario	35-44	1516	38.0 (37.0-42.0)	43	9461.34	4.54	3.29	6.12	1 (Ref.)		1 (Ref.)	1 (Ref.)
Ontario	45+	757	53.0 (49.0-59.0)	89	7251.81	12.27	9.86	15.10	1 (Ref.)		1 (Ref.)	1 (Ref.)
Ontario	Total	3650	42.0 (30.0-52.0)	197	84398.25	2.33			1 (Ref.)		1 (Ref.)	1 (Ref.)
22q11.2DS	00-17	302	*NA	*1-5	4343.66	*0.23-1.15	.	.	*0.80-4.02 (0.10-11.77)	<0.05	0.80 (0.10-6.29)	4.02 (1.38-11.77)
22q11.2DS	18-24	333	22.5 (20.5-24.0)	8	1979.60	4.04	1.74	7.96	3.21 (1.42-7.24)	0.0051		
22q11.2DS	25-34	292	30.0 (27.0-32.0)	21	1789.75	11.73	7.26	17.94	5.55 (3.22-9.56)	<.0001		
22q11.2DS	35-44	163	37.0 (37.0-42.0)	13	916.15	14.19	7.56	24.27	3.12 (1.68-5.81)	0.0003		
22q11.2DS	45+	74	51.0 (46.0-54.0)	14	569.84	24.57	13.43	41.22	2.00 (1.14-3.52)	0.0158		
22q11.2DS	Total	365	33.0 (26.5-42.5)	*57-61	9598.99	*5.94-6.35			*2.54-2.72 (1.89-3.63)	<.0001	2.54 (1.89-3.42)	2.72 (2.04-3.63)
22q11.2DS - Neither	00-17	136	*NA	*1-5	2017.39	*0.5-2.48	.	.	*1.73-8.66 (0.22-25.34)	0.1086	1.73 (0.22-13.53)	8.66 (2.96-25.34)
22q11.2DS - Neither	18-24	147	*NA	*1-5	844.88	*1.18-5.92	.	.	*0.94-4.69 (0.13-12.45)	0.9508	0.94 (0.13-6.98)	4.69 (1.77-12.45)
22q11.2DS - Neither	25-34	113	*NA	*1-5	709.17	*1.41-7.05	.	.	*0.67-3.33 (0.09-8.52)	0.0119	0.67 (0.09-4.87)	3.33 (1.30-8.52)
22q11.2DS - Neither	35-44	62	37.0 (36.0-41.0)	6	334.70	17.93	6.58	39.02	3.94 (1.68-9.27)	0.0016		
22q11.2DS - Neither	45+	27	*NA	*1-5	258.78	*3.86-19.32	.	.	*0.31-1.57 (0.04-3.88)	0.6518	0.31 (0.04-2.26)	1.57 (0.64-3.88)
22q11.2DS - Neither	Total	160	36.0 (29.0-42.0)	*15-19	4164.92	*3.6-4.56			*1.54-1.95 (0.91-3.13)	0.0124	1.54 (0.91-2.61)	1.95 (1.22-3.13)

Ontario = Ontario population comparators. 22q11.2DS = 22q11.2 deletion syndrome, i.e., 22q-cases. 22q11.2DS – Neither = the subgroup of 22q-cases with neither major congenital heart disease or schizophrenia. Age group is in years N=number. IQR = inter-quartile range. PYAR = person-years at risk, calculated as any risk time observed within the age group, even if beginning in the middle or towards the end of a period. IR = incidence rate. * Small cell data suppressed as ICES policies prohibit the release of small cell data (<6) or other corresponding data allowing back calculation.

A confidence interval not including 1.0 was considered statistically significant. P-values for the incidence rate ratios were calculated using the Wald chi-square statistic.

Table S10. Incidence of Stroke/transient ischemic attack

Group	Age group	N at start of age interval	Median (IQR) age at diagnosis	New cases	PYAR	Incidence per 1000 person years	IR lower	IR upper	Incidence rate ratio (95%CI)	Incidence rate ratio (95%CI) if 1 or lower value of range provided	Incidence rate ratio (95%CI) if 5 or higher value of range provided	Incidence rate ratio (95%CI) if 22q have 1 or lower value of range provided, and controls have 5 or higher value of range provided	Incidence rate ratio (95%CI) if controls have 1 or lower value of range provided, and 22q have 5 or higher value of range provided
Stroke/TIA (Karen Tu)													
Ontario	00-17	2482	*NA	*1-5	34982.08	*0.03-0.14	.	.	1 (Ref.)	1 (Ref.)	1 (Ref.)	1 (Ref.)	1 (Ref.)
Ontario	18-24	2902	*NA	*1-5	16737.15	*0.06-0.30	.	.	1 (Ref.)	1 (Ref.)	1 (Ref.)	1 (Ref.)	1 (Ref.)
Ontario	25-34	2814	*NA	*1-5	16242.79	*0.06-0.31	.	.	1 (Ref.)	1 (Ref.)	1 (Ref.)	1 (Ref.)	1 (Ref.)
Ontario	35-44	1516	39.0 (37.0-43.0)	11	9727.18	1.13	0.56	2.02	1 (Ref.)	1 (Ref.)	1 (Ref.)	1 (Ref.)	1 (Ref.)
Ontario	45+	757	58.0 (51.0-65.0)	22	8160.60	2.70	1.69	4.08	1 (Ref.)	1 (Ref.)	1 (Ref.)	1 (Ref.)	1 (Ref.)
Ontario	Total	3650	46.0 (35.0-58.0)	42	85849.80	0.49			1 (Ref.)	1 (Ref.)	1 (Ref.)	1 (Ref.)	1 (Ref.)
22q11.2DS	00-17	302	0.0 (0.0-11.0)	9	4259.94	2.11	0.97	4.01	*14.78-73.91 (4.95-583.35)	73.91 (9.36-583.35)	14.78 (4.95-44.11)		
22q11.2DS	18-24	333	*NA	*1-5	1963.03	*0.51-2.55	.	.	*1.71-42.63 (0.20-364.90)	8.53 (0.53-136.31)	8.53 (2.47-29.45)	1.71 (0.20-14.60)	42.63 (4.98-364.90)
22q11.2DS	25-34	292	*NA	*1-5	1881.86	*0.53-2.66	.	.	*1.73-43.16 (0.20-369.39)	8.63 (0.54-137.99)	8.63 (2.50-29.81)	1.73 (0.20-14.78)	43.16 (5.04-369.39)
22q11.2DS	35-44	163	*NA	*1-5	1045.54	*0.96-4.78	.	.	*0.85-4.23 (0.11-12.17)	0.85 (0.11-6.55)	4.23 (1.47-12.17)		
22q11.2DS	45+	74	*NA	*1-5	715.13	*1.40-6.99	.	.	*0.52-2.59 (0.07-6.85)	0.52 (0.07-3.85)	2.59 (0.98-6.85)		
22q11.2DS	Total	365	22.0 (0.0-44.0)	19	9865.49	1.93			3.94 (2.29-6.77)				
22q11.2DS - Neither	00-17	136	*NA	*1-5	2019.81	*0.5-2.48	.	.	*3.46-86.60 (0.40-741.23)	17.32 (1.08-276.90)	17.32 (5.01-59.83)	3.46 (0.40-29.65)	86.60 (10.12-741.23)
22q11.2DS - Neither	18-24	147	*NA	*1-5	845.39	*1.18-5.91	.	.	*3.96-98.99 (0.46-847.31)	19.80 (1.24-316.52)	19.80 (5.73-68.39)	3.96 (0.46-33.89)	98.99 (11.57-847.31)
22q11.2DS - Neither	25-34	113	. (-.)	0	711.22	0.00	.	5.19	...				
22q11.2DS - Neither	35-44	62	. (-.)	0	365.05	0.00	.	10.11	...				
22q11.2DS - Neither	45+	27	*NA	*1-5	305.36	0.00	.	12.08	*1.21-6.07 (0.16-16.04)	1.21 (0.16-9.01)	6.07 (2.30-16.04)		
22q11.2DS - Neither	Total	160	*NA	*1-5	4246.83	*0.24-1.18			*0.48-2.41 (0.07-6.08)	0.48 (0.07-3.50)	2.41 (0.95-6.08)		

Ontario = Ontario population comparators. 22q11.2DS = 22q11.2 deletion syndrome, i.e., 22q-cases. 22q11.2DS – Neither = the subgroup of 22q-cases with neither major congenital heart disease or schizophrenia. Age group is in years N=number. IQR = inter-quartile range. PYAR = person-years at risk, calculated as any risk time observed within the age group, even if beginning in the middle or towards the end of a period. IR = incidence rate. * Small cell data suppressed as ICES policies prohibit the release of small cell data (<6) or other corresponding data allowing back calculation. A confidence interval not including 1.0 was considered statistically significant.

Table S11. Cost analysis up to Aug 2023

Group	N	Relative Ratio	95%CI	P
**Age group 0 to 17 years**				
**Matched Ontario controls**	2062	Ref.		
**People with 22q11.2DS**	239	9.43	8.31-10.69	<0.0001
**Age group 18 to 24 years**				
**Matched Ontario controls**	2594	Ref.		
**People with 22q11.2DS**	288	7.97	6.88-9.22	<0.0001
**Age group 25 to 34 years**				
**Matched Ontario controls**	2426	Ref.		
**People with 22q11.2DS**	248	6.99	5.95-8.22	<0.0001
**Age group 35 to 44 years**				
**Matched Ontario controls**	1331	Ref.		
**People with 22q11.2DS**	142	10.03	8.26-12.20	<0.0001
**Age group 45+ years**				
**Matched Ontario controls**	757	Ref.		
**People with 22q11.2DS**	74	7.66	5.73-10.25	<0.0001

N=number of individuals in the age group for which costs were calculated. CI = confidence interval. A confidence interval not including 1.0 was considered statistically significant. P-values for the relative ratios were calculated using the Wald chi-square statistic.

Table S12. Cost analysis up to Aug 2023 by subgroup

Subgroup		N	Relative Ratio	95%CI	P
**Age group 0 to 17 years**					
**Matched Ontario controls**				Ref.	
	**CHD**	81	16.84	13.88-20.43	<0.0001
**People with 22q11.2DS**	**Neither**	114	5.52	4.94-6.56	<0.0001
	**Schizophrenia**	44	6.29	4.79-8.25	<0.0001
**Age group 18 to 24 years**					
**Matched Ontario controls**				Ref.	
	**CHD**	96	4.23	3.86-6.29	<0.0001
**People with 22q11.2DS**	**Neither**	132	3.71	2.99-4.60	<0.0001
	**Schizophrenia**	60	22.01	16.82-28.81	<0.0001
**Age group 25 to 34 years**					
**Matched Ontario controls**				Ref.	
	**CHD**	83	3.97	3.02-5.21	<0.0001
**People with 22q11.2DS**	**Neither**	94	2.24	1.71-2.93	<0.0001
	**Schizophrenia**	71	16.79	12.94-21.78	<0.0001
**Age group 35 to 44 years**					
**Matched Ontario controls**				Ref.	
	**CHD**	39	6.24	4.36-8.91	<0.0001
**People with 22q11.2DS**	**Neither**	53	5.07	3.70-6.94	<0.0001
	**Schizophrenia**	50	17.94	13.53-23.79	<0.0001
**Age group 45+ years**					
**Matched Ontario controls**				Ref.	
	**CHD**	10	3.95	1.76-8.88	0.0009
**People with 22q11.2DS**	**Neither**	27	2.51	1.53-4.10	0.0002
	**Schizophrenia**	37	12.56	8.57-18.42	<0.0001

N=number of individuals in the age group for which costs were calculated. CI = confidence interval. CHD = major congenital heart disease group. Neither = neither major CHD nor schizophrenia group. A confidence interval not including 1.0 was considered statistically significant. P-values for the relative ratios were calculated using the Wald chi-square statistic.

Table S13. Cost sensitivity analysis, restriction to pre-pandemic (March 2020)

Group	Relative Ratio	95%CI	P
**Age group 0 to 17 years**			
**Matched Ontario controls**	Ref.		
**People with 22q11.2DS**	9.38	8.27-10.64	< 0.0001
**Age group 18 to 24 years**			
**Matched Ontario controls**	Ref.		
**People with 22q11.2DS**	8.08	6.95-9.40	< 0.0001
**Age group 25 to 34 years**			
**Matched Ontario controls**	Ref.		
**People with 22q11.2DS**	7.32	6.14-8.73	< 0.0001
**Age group 35 to 44 years**			
**Matched Ontario controls**	Ref.		
**People with 22q11.2DS**	8.71	7.07-10.74	< 0.0001
**Age group 45+ years**			
**Matched Ontario controls**	Ref.		
**People with 22q11.2DS**	7.90	5.75-10.87	< 0.0001

CI = confidence interval. A confidence interval not including 1.0 was considered statistically significant. P-values for the relative ratios were calculated using the Wald chi-square statistic.

Table S14. Cost sensitivity analysis, restriction to pre-pandemic (March 2020), by subgroup

Subgroup		Relative Ratio	95%CI	P
**Age group 0 to 17 years**				
**Matched Ontario controls**			Ref.	
	**CHD**	16.73	13.79-20.31	<0.0001
**People with 22q11.2DS**	**Neither**	5.50	4.63-6.54	<0.0001
	**Schizophrenia**	6.26	4.77-8.23	<0.0001
**Age group 18 to 24 years**				
**Matched Ontario controls**			Ref.	
	**CHD**	4.29	3.34-5.51	<0.0001
**People with 22q11.2DS**	**Neither**	3.54	2.81-4.44	<0.0001
	**Schizophrenia**	22.07	16.98-28.69	<0.0001
**Age group 25 to 34 years**				
**Matched Ontario controls**			Ref.	
	**CHD**	5.10	3.80-6.85	<0.0001
**People with 22q11.2DS**	**Neither**	2.50	1.85-3.38	<0.0001
	**Schizophrenia**	15.39	11.68-20.28	<0.0001
**Age group 35 to 44 years**				
**Matched Ontario controls**			Ref.	
	**CHD**	7.61	5.07-11.41	<0.0001
**People with 22q11.2DS**	**Neither**	4.80	3.40-6.77	<0.0001
	**Schizophrenia**	12.83	9.60-17.16	<0.0001
**Age group 45+ years**				
**Matched Ontario controls**			Ref.	
	**CHD**[a]	2.43	0.86-7.18	0.0906
**People with 22q11.2DS**	**Neither**	1.96	1.16-3.33	0.0123
	**Schizophrenia**	13.68	9.10-20.57	<0.0001

CI = confidence interval. CHD = major congenital heart disease group. Neither = neither major CHD nor schizophrenia group.
[a]There were only 6 individuals in this subgroup. A confidence interval not including 1.0 was considered statistically significant. P-values for the relative ratios were calculated using the Wald chi-square statistic.

Table S15. Cost sensitivity analysis, by time since diagnosis subgroups

Subgroup		N	Relative Ratio	95%CI
**Age group 0 to 17 years**				
**Matched Ontario controls**			Ref.	
	**Before dx**	*238-234	9.18	8.09-10.42
**People with 22q11.2DS**	**First 5 years**	*1-5		
	**>5 y**	*1-5		
**Age group 18 to 24 years**				
**Matched Ontario controls**			Ref.	
	**Before dx**	63	9.97	7.47-13.31
**People with 22q11.2DS**	**First 5 years**	32	16.63	11.43-24.20
	**>5 y**	193	5.94	4.98-7.07
**Age group 25 to 34 years**				
**Matched Ontario controls**			Ref.	
	**Before dx**	57	8.30	6.13-11.22
**People with 22q11.2DS**	**First 5 years**	27	12.03	7.96-18.18
	**>5 y**	164	5.60	4.60-6.82
**Age group 35 to 44 years**				
**Matched Ontario controls**			Ref.	
	**Before dx**	36	11.43	8.14-16.06
**People with 22q11.2DS**	**First 5 years**	22	8.88	5.75-13.72
	**>5 y**	84	9.00	7.59-12.40
**Age group 45+ years**				
**Matched Ontario controls**			Ref.	
	**Before dx**	21	14.88	9.30-23.82
**People with 22q11.2DS**	**First 5 years**	12	5.16	2.63-10.15
	**>5 y**	41	4.04	2.69-6.08

CI = confidence interval. Before dx = Before molecular diagnosis, First 5 years = first 5 years after molecular diagnosis, >5 y =6 years or more after molecular diagnosis. * Small cell data suppressed as ICES policies prohibit the release of small cell data (<6) or other corresponding data allowing back calculation. A confidence interval not including 1.0 was considered statistically significant.

Table S16. Hospitalizations

	Andersen-Gill/Marginal Means and Rates Model Crude RR (95%CI)
**Hospitalizations from 18 years on among those with follow-up**	
Ontario Controls	1 (Ref.)
22q Cases	3.90 (3.59-4.23)
Schizophrenia	7.14 (6.46-7.88)
CHD	2.85 (2.47-3.28)
Neither	2.08 (1.78-2.44)

CI = Confidence interval. CHD = major congenital heart disease. Hospitalizations include both acute care and mental health hospitalization records from the discharge abstract database (DAD) and Ontario Mental Health Reporting System (OMHRS) databases (see e-Table 1). The counting process was used to program the start and stop time for hospitalizations. Relative rate of hospitalizations was estimated using the recurrent event framework. The time scale used was age. See text for more details. A confidence interval not including 1.0 was considered statistically significant. Confidence intervals were calculated based on a robust variance estimator accounting for clustering at the level of the individual.

Figure S1. A and B. Study timelines.

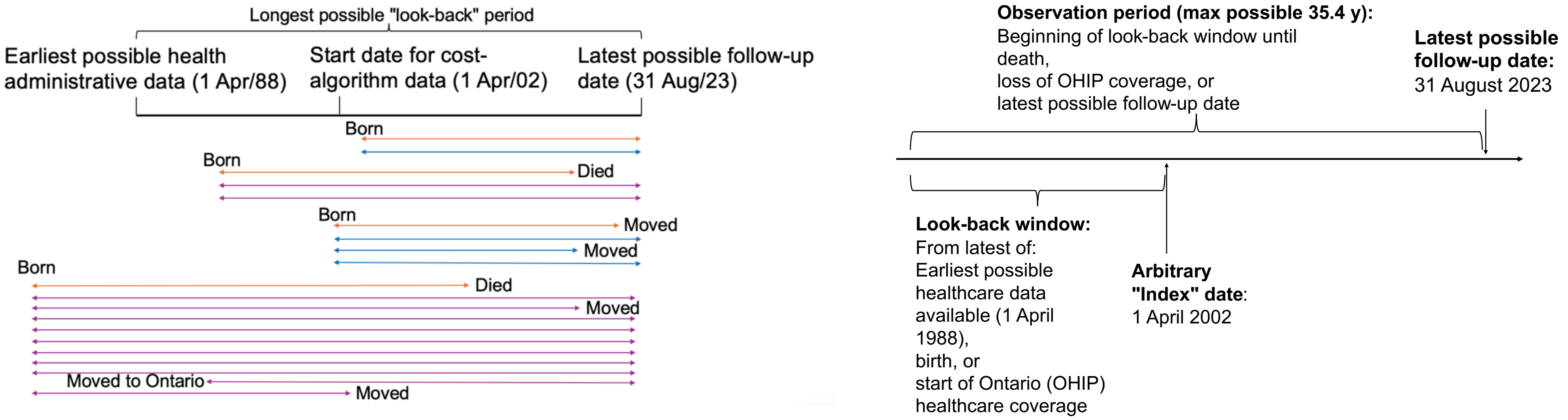

OHIP = Ontario Health Insurance Plan. See e-Table 1 for more information on data sources. 1 April 2002 represents date of first availability of healthcare cost data in ICES. All 22q-cases and population-comparators were required to be alive and have OHIP eligibility/coverage as an adult (i.e., aged ≥ 18 y), and to have OHIP eligibility/coverage as at 1 April 2002. The prior cohort study involving the originating linked 22q-case cohort and population-comparators focused on overall cumulative healthcare costs, with no data beyond 31 March 2020, and no incidence data.[10]

Figure S2. Incidence of non-validated cardiovascular and other Charlson conditions.

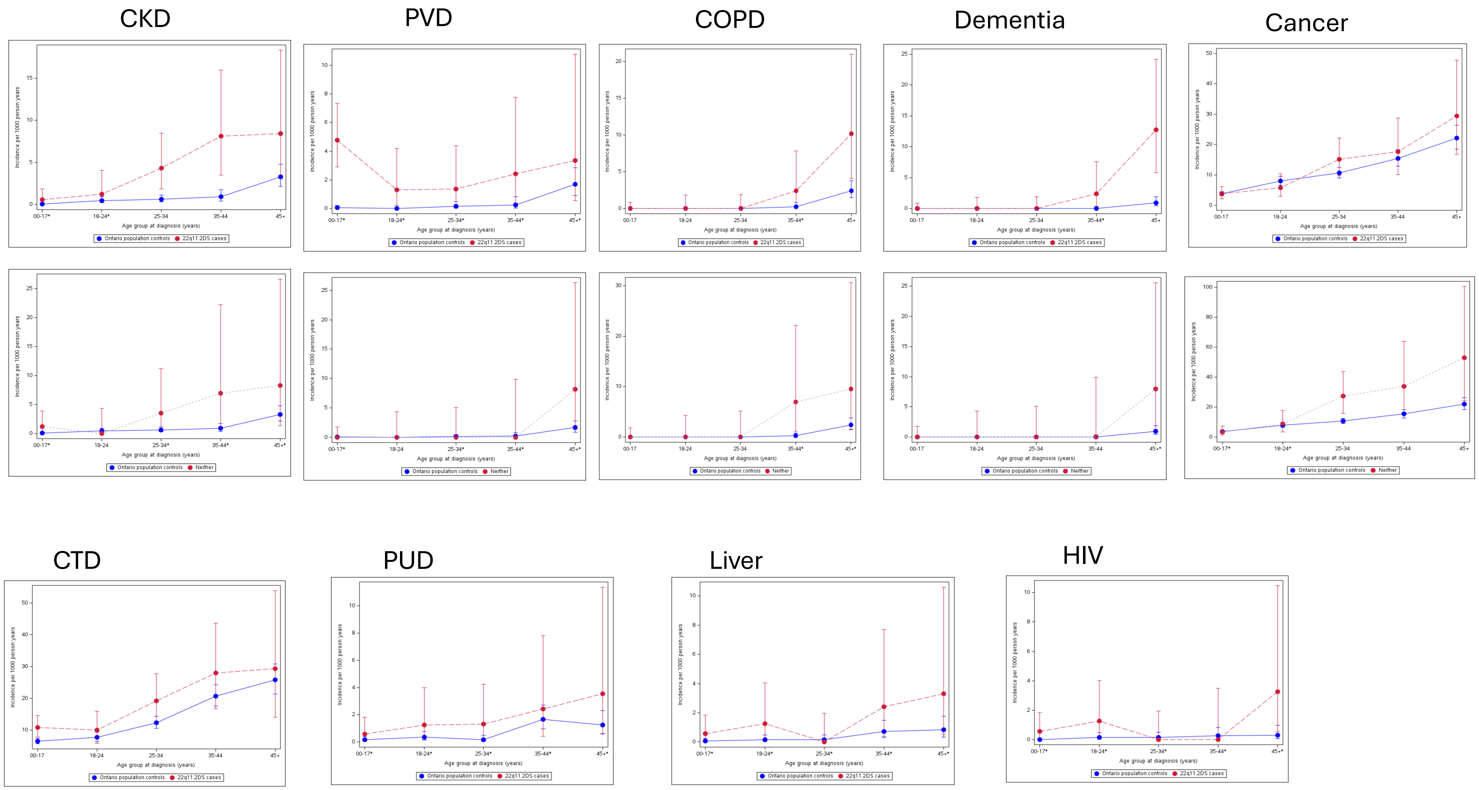

CKD = Chronic kidney disease/renal failure. PVD = peripheral vascular disease. COPD = chronic obstructive pulmonary disease. CTD = rheumatoid arthritis, connective tissue disorders and osteoarthritis. PUD = peptic ulcer disease. Liver = liver disease. (see e-Table 2 for more details on definitions of each condition). The graphs display for each condition the incidence (first occurrence of the condition) per 1,000 person-years (Y axis) across five age categories (X axis); with

varying numbers on the Y axis. Groups compared in each graph are labeled. 22q-cases (red) and general population-comparators (blue); or 22q-case subgroup "Neither" (22q-case individuals with neither major CHD nor schizophrenia; red) vs the same general population-comparators (blue).
An asterisk (*) indicates that the incidence rate ratio and confidence interval were estimated due to small cell sizes. See main text for further details.

Figure S3. Incidence of six conditions known to be associated with 22q11.2 microdeletion.

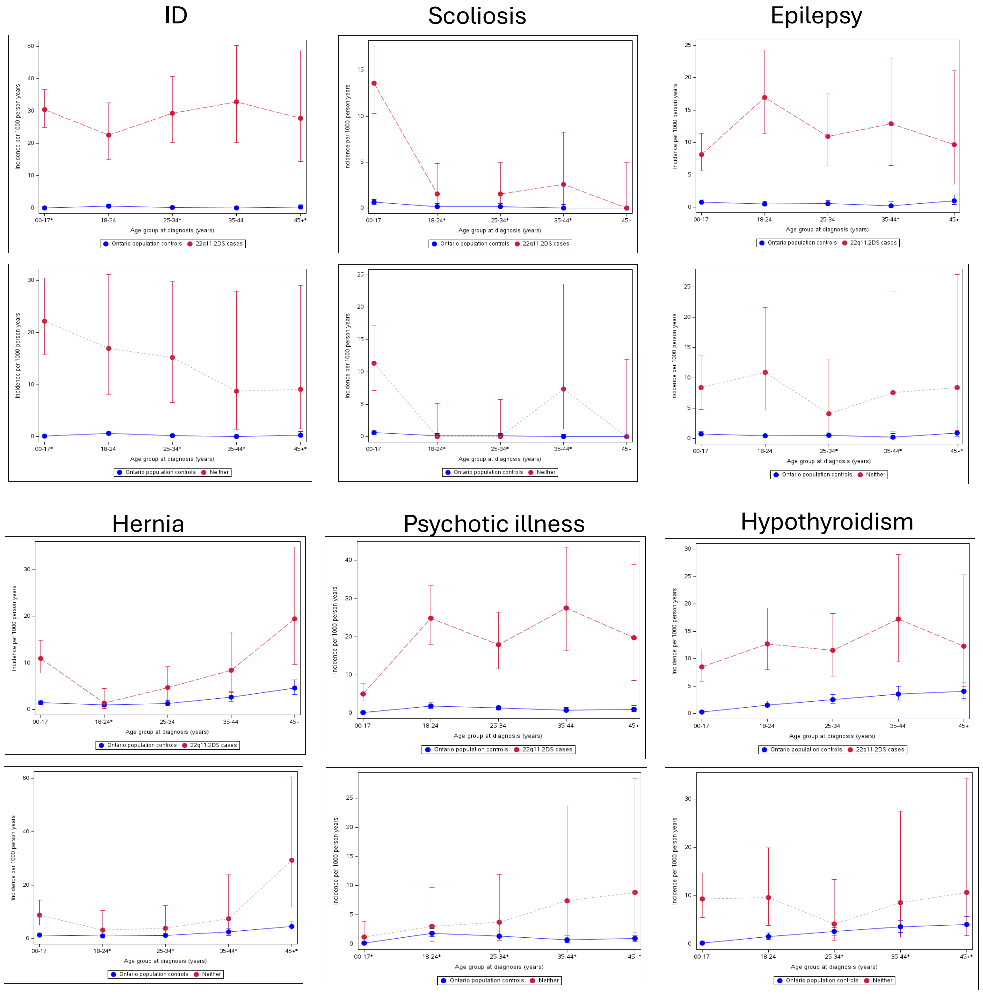

ID =intellectual disability. (see e-Table 2 for more details on definitions of each condition). The graphs display for each condition the incidence (first occurrence of the condition) per 1,000 person-years (Y axis) across five age categories (X axis); with varying numbers on the Y axis. Groups compared in each graph are labeled. 22q-cases (red) and general population-comparators (blue); or 22q-case subgroup "Neither" (22q-case individuals with neither major CHD nor schizophrenia; red) vs the same general population-comparators (blue).
An asterisk (*) indicates that the incidence rate ratio and confidence interval were estimated due to small cell sizes. See main text for further details.

Figure S4. Cost sensitivity analysis, by time since diagnosis subgroups

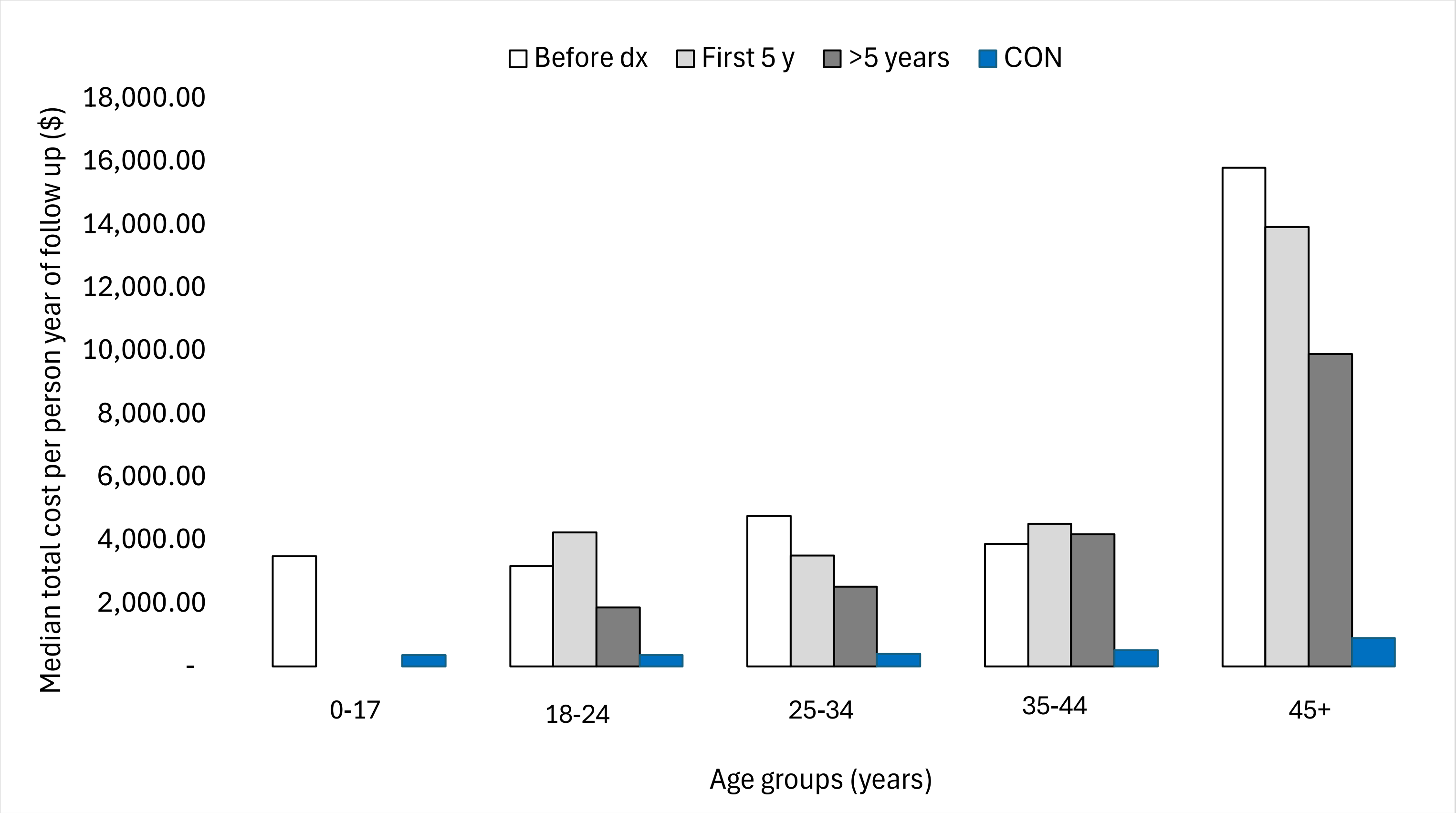

The time since molecular genetic diagnosis was calculated to be at the beginning of the follow-up period for each of five age groups. Note that for some individuals the timing may have been in the middle of the follow-up period (e.g., if the individual moved to the province at age 21, or turned 18 before April 1 2002 (date of the inception of the cost algorithm).
N for each age group is provided in e-Table 15.
For each age group, for the 22q-case individuals white bars indicate costs before genetic diagnosis, pale grey bars indicate costs in the first five years after genetic diagnosis, and dark grey bars indicate costs over 5 years after genetic diagnosis; blue bars indicate costs for the general population-comparators.

References

1. Wodchis WP BK, Nikitovic M, McKillop I. *Guidelines on Person-Level Costing Using Administrative Databases in Ontario.* Toronto, Ontario 2013.
2. Tu K, Campbell NR, Chen ZL, Cauch-Dudek KJ, McAlister FA. Accuracy of administrative databases in identifying patients with hypertension. *Open Med.* 2007;1(1):e18-26.
3. Hux JE, Ivis F, Flintoft V, Bica A. Diabetes in Ontario: determination of prevalence and incidence using a validated administrative data algorithm. *Diabetes Care.* 2002;25(3):512-516.
4. Chau E RL, Mondor L, Wodchis WP. Association between continuity of care and subsequent diagnosis of multimorbidity in Ontario, Canada from 2001–2015: A retrospective cohort study. *PLOS ONE.* 2021;16(3).
5. Austin PC, Daly PA, Tu JV. A multicenter study of the coding accuracy of hospital discharge administrative data for patients admitted to cardiac care units in Ontario. *Am Heart J.* 2002;144(2):290-296.
6. Schultz SE, Rothwell DM, Chen Z, Tu K. Identifying cases of congestive heart failure from administrative data: a validation study using primary care patient records. *Chronic Dis Inj Can.* 2013;33(3):160-166.
7. Tu K, Wang M, Young J, et al. Validity of Administrative Data for Identifying Patients Who Have Had a Stroke or Transient Ischemic Attack Using EMRALD as a Reference Standard. *Canadian Journal of Cardiology.* 2013;29(11):1388-1394.
8. Gershon AS, Wang C, Guan J, Vasilevska-Ristovska J, Cicutto L, To T. Identifying individuals with physcian diagnosed COPD in health administrative databases. *Copd.* 2009;6(5):388-394.
9. Jaakkimainen RL, Bronskill SE, Tierney MC, et al. Identification of Physician-Diagnosed Alzheimer's Disease and Related Dementias in Population-Based Administrative Data: A Validation Study Using Family Physicians' Electronic Medical Records. *J Alzheimers Dis.* 2016;54(1):337-349.
10. Malecki SL, Heung T, Wodchis WP, et al. Young adults with a 22q11.2 microdeletion and the cost of aging with complexity in a population-based context. *Genet Med.* 2024;26(5):101088.
11. Brown HK, Ray JG, Chen S, et al. Association of Preexisting Disability With Severe Maternal Morbidity or Mortality in Ontario, Canada. *JAMA Netw Open.* 2021;4(2):e2034993.
12. Kurdyak P, Lin E, Green D, Vigod S. Validation of a Population-Based Algorithm to Detect Chronic Psychotic Illness. *Can J Psychiatry.* 2015;60(8):362-368.
13. Tonelli M, Wiebe N, Fortin M, et al. Methods for identifying 30 chronic conditions: application to administrative data. *BMC Med Inform Decis Mak.* 2015;15:31.
